# Mapping of Iranian midwifery curriculum according to the International Confederation of midwives competencies

**DOI:** 10.1186/s12909-023-04755-7

**Published:** 2023-10-24

**Authors:** Somayeh Abdolalipour, Sakineh Mohammad-Alizadeh-Charandabi, Farah Babaey, Leila Allahqoli, Reza Ghaffari, Mojgan Mirghafourvand

**Affiliations:** 1 Department of Midwifery, Faculty of Nursing and Midwifery, Tabriz University of Medical Sciences, Tabriz, IR Iran; 2 Social Determinants of Health Research Center, Tabriz University of Medical Sciences, Tabriz, IR Iran; 3 Midwifery Department, Ministry of Health and Medical Education, Tehran, Iran; 4 Department of Medical Education, Education Development Center, Tabriz University of Medical Sciences, Tabriz, IR Iran

**Keywords:** Midwifery education, Curriculum, International Confederation of Midwives, Competence, Educational standards

## Abstract

**Background:**

Evaluating the curriculum based on its success rate in preparing skilled midwives proficient in performing professional skills is a fundamental component of the midwifery education system. This study aimed to evaluate the content, strengths, and weaknesses of the midwifery curriculum in Iran based on the most recent ICM midwifery education standards in all competence areas, as well as to obtain expert feedback on the necessary courses or lessons for the curriculum using the Delphi method.

**Methods:**

This research was conducted in two phases: comparative analysis and the Delphi method. In the comparative analysis, the curriculum mapping tool was used to compare Iran’s midwifery curriculum for bachelor’s degrees to the international standards for midwifery education proposed by ICM in 2019 by a four-point Likert scale (adequate- relatively adequate- relatively inadequate- inadequate). Two individuals evaluated the curriculum independently for the presence of theoretical and clinical courses for attaining each relevant competency. In case of disagreement, the opinion of a third person was used. After identifying the academic deficiencies and weaknesses of the curriculum, the Delphi technique was used with the cooperation of the midwifery board members and directors of midwifery groups from across the country to collect feedback about new courses or lessons that need to be incorporated into the curriculum.

**Results:**

After a comparative analysis, 24 out of 315 essential competencies for ICM in the midwifery curriculum were found to be inadequate or relatively inadequate based on the three experts’ opinions after reviewing the programmatic courses and lessons in the curriculum. In 79.5% of the knowledge area and 71.6% of the skill area, the curriculum for midwifery in Iran corresponded to ICM essential competencies. After surveying expert members during multiple Delphi rounds, the members agreed to add some lessons to the midwifery curriculum, design a new course, and hold related workshops to cover the competencies identified as inadequate or relatively inadequate in the comparative analysis.

**Conclusion:**

The Iranian midwifery curriculum for acquiring 24 items of ICM essential competencies was deemed inadequate or relatively inadequate. Therefore, it seems in addition to revising Iran’s midwifery curriculum following ICM competencies, providing midwifery policymakers with infrastructure and additional support to develop and implement effective midwifery training programs is necessary to ensure that midwives are trained and equipped with the necessary competencies for practice.

**Supplementary Information:**

The online version contains supplementary material available at 10.1186/s12909-023-04755-7.

## Background

Midwives have contributed significantly to reducing maternal and newborn mortality, and they are required in all settings and countries to provide high-quality maternal and newborn care. Midwives have played an important role in achieving the Millennium Development Goals. In addition, their participation is crucial for achieving Sustainable Development Goals (SDGs), such as SDG 3, which aims to further reduce maternal and infant morbidity and mortality [1]. The International Confederation of Midwifery (ICM) defines a midwife as a responsible and accountable person who provides women with the necessary support, care, and advice throughout their pregnancies, deliveries, and postpartum periods, and cares for their infants.” This care includes taking preventive measures, promoting normal vaginal childbirth, diagnosing issues affecting mothers and children, providing access to medical care and other appropriate assistance, and participating in emergency procedures [2].

Evolving knowledge and advancements in education and learning technology pose challenges for accredited professional programs. Therefore, evaluating the curriculum based on its success rate in preparing skilled midwives proficient in performing professional skills is a fundamental component of the midwifery education system. The World Health Organization (WHO), The United Nations Population Fund (UNFPA), and ICM organizations currently support such assessments in some countries [3]. The ICM suggests standard training programs, and midwives should be trained according to these programs [4]. This confederation supports core competencies for the midwifery profession and global standards for their education to establish global standards for midwifery education and practice [5].

In August 2019, an additional update was implemented to promote and enhance the skills component of the competencies. In addition, some competencies underwent a minor revision in October 2019 to further emphasize the midwife’s role in preventing, diagnosing, and managing complications. These competencies were divided into four interrelated categories: (1) General competencies (48 items for knowledge and 68 items for skills/behaviors): these competencies are necessary for the midwife’s autonomy and responsibility as a healthcare provider, her interactions with patients and other healthcare professionals, and her caregiving duties; these competencies apply to every aspect of midwifery practice. (2) Competencies needed for pre-pregnancy and pregnancy care (37 items for knowledge and 45 items for skills/behaviors): this class requires competencies in assessing the health of the mother and fetus, promoting health and well-being, diagnosing pregnancy-related complications, and caring for women who experience unplanned pregnancies. (3) Competencies needed for care during labor and childbirth (19 items for knowledge and 35 items for skills/behaviors): These competencies are needed to evaluate and care for women during labor and childbirth, support physiological processes and safe delivery, provide immediate care for the newborn, diagnose complications in the mother or newborn, stabilize emergency conditions, and, if necessary, referral. (4) Competencies needed for the continuous care of mothers and infants (28 items for knowledge and 35 items for skills/behaviors): Family planning services, health education, support for breastfeeding, diagnosis of complications, stabilization and referral in emergencies, and continuous assessment of mother and infant health [6].

Midwifery education in Iran is carried out at the bachelor’s, master’s, and PhD levels. Midwifery students are admitted through a national exam. They must pass 130 theoretical and clinical courses in four years to obtain a bachelor’s degree. These courses include 20 general courses, 20 basic sciences courses, 72 specialized courses, 2 optional specialized courses, and 16 internship courses. Students enter clinical settings after the first semester under the supervision of clinical instructors. The Ministry of Health and Medical Education of Iran has designed midwifery curricula at all levels for universities in the country. The midwifery curriculum was designed by the Ministry of Health and Medical Education of Iran at all levels and a significant part of it is devoted to the acquisition of clinical skills that are necessary for professional midwifery activities [7].

Despite the efforts made to improve midwifery education in Iran, studies do not show much success in achieving the envisioned objectives for midwifery education. Based on evidence, the professional capabilities of students have decreased compared to the previous decades [8, 9]. The results of a qualitative study showed that an inappropriate educational atmosphere and an inefficient curriculum are the main issues of midwifery education in Iran [10]. Moreover, studies report students lack skills and efficiency in clinical settings despite their adequate theoretical knowledge [11, 12]. Weakness of educational programs such as inefficient practical skills is the most common opinion from the point of view of midwifery instructors and graduates in another qualitative study [13]. Bahri et al. conducted a comparative study in 2018. They only compared the “Pregnancy Care” section of Iran’s midwifery curriculum to the international standards of ICM 2013. Iranian Midwifery Curriculum met the ICM standards except for two items (“Signs of female genital cutting and effects on reproductive health” and “Normal limits of results from community-relevant laboratory tests commonly performed in pregnancy”). The authors recommended changes in the midwifery curriculum and more clinical training hours [14]. A greater number of general courses compared to specialized courses in the curriculum is another challenge of the midwifery curriculum from the midwifery instructors’ point of view [15].

Based on studies conducted in Iran, the midwifery curriculum faces some challenges from the perspective of midwifery students, graduates, and instructors. Identifying appropriate strategies to address these challenges for strengthening midwives, requires understanding the current state of the midwifery profession, including its gaps and difficulties [16]. An improved professional framework for midwifery education could strengthen the development of midwifery education programs. Based on the ICM, if every country implemented these professional standards, it would see higher quality midwifery education and thus improved reproductive health for women, newborns, and their families [17]. Therefore, this study aimed to evaluate the content of the midwifery curriculum in Iran based on the most recent international standards of ICM midwifery education in all fields of competencies. In this regard, the Delphi method was used to obtain expert feedback regarding the new lessons required for the curriculum.

**Study objectives**.

1. To compare midwifery curriculum contents with essential competencies of ICM for midwifery, 2019.
2. To provide suggestions by experts using the Delphi method to make the midwifery curriculum more compatible with essential competencies of ICM for midwifery.

## Methods

This research was conducted in two phases: comparative analysis and recommending modifications using the Delphi method.

### The first phase

In the first phase, a comparative study was conducted to check the compatibility of the midwifery curriculum for the bachelor’s degree with the international standards of midwifery education proposed by ICM in 2019. For this purpose, the Curriculum Mapping tool was utilized. The Curriculum Mapping tool which was invented in 1970 for the first time, offers many benefits to curriculum planners, including the chance to become more familiar with the curriculum structures and relationships that can align the student’s learning outcomes to the rest of the curriculum [18]. The implication of this tool in this study is at the “course level” which is mapping ICM competencies in both forms of knowledge and skills/behaviors across the entire courses of the curriculum. Professional competencies (student learning outcomes) that consist of 315 items are identified along one axis and programmatic courses along the other axis of this tool. The column titled ‘adequacy’ in this tool was used to rate the degree of course compliance with the competencies by a four-point Likert scale (adequate- relatively adequate- relatively inadequate- inadequate) [14]. Two individuals evaluated the curriculum independently for the presence of theoretical and clinical courses for attaining each relevant competency and the necessity of adding a new course or lesson to the curriculum to acquire a specific competency. In the case of full coverage of each competency item by the programmatic courses, it was rated as “adequate” and in case of lack of content, it was rated as “inadequate. In other cases, by asking for the opinion of a third party, the rating was done as “relatively adequately” or “relatively adequate” (Appendix, Table).

### The second phase

After identifying the academic deficiencies and weaknesses of the curriculum and having them confirmed by representatives of midwifery board members and directors of midwifery groups from across the country, the Delphi technique (Opinions from health managers, policymakers, and subject-matter experts) [19] was used to collect feedback about new courses or lessons that need to be incorporated into the curriculum.

### The Delphi steps were as follows

#### The first step

First, the experts were identified, and then, after being informed of the purpose and methodology, they were invited to participate. Individuals with educational experience in midwifery and knowledge of issues related to the undergraduate curriculum for midwifery were selected using purposive sampling. The panel of experts consisted of nine academics from different Iranian universities (Tabriz, Tehran, Mashhad, and Isfahan) and one facilitator (Ph.D. student in midwifery). It should be noted that one of the experts contributing to this study is the Minister of Health’s advisor on midwifery matters. In addition, the majority of other involved experts are members of the Ministry of Health’s specialized midwifery board, which is responsible for revising the midwifery curriculum at the bachelor’s, master’s, and Ph.D. levels. The profile of experts who participated in the Delphi phase is displayed in Table 1. All invited individuals consistently participated in all Delphi rounds. In the first round, opinion polling sessions were conducted. The second and third rounds were conducted individually via email. A consensus was reached through a group discussion.

**Table 1:** Profiles of expert members in the Delphi phase of the study

Number	Affiliation	Scientific degree	Midwifery board member
**1**	Tehran University of Medical Sciences	Assistant Professor	√
**2**	Tehran University of Medical Sciences	Associate Professor	√
**3**	Shahid Beheshti University of Medical Sciences	Associate Professor	√
**4**	Mashhad University of Medical Sciences	Associate Professor	√
**5**	Rasht University of Medical Sciences	Professor	√
**6**	Tabriz University of Medical Sciences	Professor	√
**7**	Tabriz University of Medical Sciences	Professor	√
**8**	Tabriz University of Medical Sciences	Assistant Professor	
**9**	Isfahan University of Medical Sciences	Professor	√

#### The second step

At this stage, the facilitator provided each expert with the parts of the curriculum determined to be inadequate or relatively inadequate based on a comparison to the ICM competencies. Based on the ICM competencies, they were asked to provide written comments on the weaknesses and academic deficiencies in the midwifery curriculum for a bachelor’s degree and to suggest courses or lessons that address these issues.

#### The third step

Expert opinions were gathered regarding the flaws, weaknesses, and new lessons or courses required to address these deficiencies. Answers that shared similar concepts were merged, and duplicates were excluded. The experts’ opinions and suggestions were provided to them once more. They were asked to express their views on the new lessons, their compliance with the ICM competencies, and their applicability on a scale from fully agree, agree, disagree, completely disagree, and inapplicable. In addition, they were requested to provide descriptive comments for each item, if necessary. A consensus was deemed to exist when there was 85% or more agreement [20]. The items that did not meet the 85% consensus requirement in the earlier step were once more given to the experts for review and, if necessary, adjustment. Experts were also provided with additional information, including the duration and location of the training, the overall and specific objectives, the content outlines, the references used, and a comprehensive evaluation of the new course and lessons added.

#### The fourth step

The responses from the third step were collected, and the level of expert agreement was analyzed. The items with less than 50% consensus were removed, and the items with 50–85% consensus were again provided to the expert members for further discussion [20].

## Results

### Comparative analysis

After conducting a comparative analysis, it was determined that 24 out of 315 of the ICM competencies in the midwifery curriculum were inadequate or relatively inadequate (Table 2). Table 3 displays the frequency distribution of conformity of Iran’s midwifery curriculum in the areas of knowledge and skills with the four competence areas determined by ICM.

**Table 2:** Frequency of Iranian midwifery curriculum conformity in the areas of knowledge and skills with the ICM competencies

Competency Adequacy	General competencies		Pre-pregnancy and antenatal		Care during labor and birth		Ongoing care of women and newborns		Total	
	Knowledge (48 Items)	Skills & Behaviors (68 Items)	Knowledge (37 Items)	Skills & Behaviors (45 Items)	Knowledge (19 Items)	Skills & Behaviors (35 Items)	Knowledge (28 Items)	Skills & Behaviors (35 Items)	Knowledge (132 Items)	Skills & Behaviors (183 Items)
**Adequate** Number (%)	38 (79.2)	43 (62.3)	29 (78.4)	27 (61.3)	15 (78.9)	32 (91.4)	23 (78.6)	29 (82.9)	105 (79.5)	131 (71.6)
**Relatively adequate** Number (%)	6 (12.5)	20 (29)	5 (13.5)	12 (27.3)	3 (15.8)	3 (8.6)	5 (21.4)	1 (2.9)	19 (14.4)	36 (19.7)
**Relatively inadequate** Number (%)	-	1 (1.5)	1 (2.7)	-	-	-	-	2 (5.7)	1 (0.8)	3 (1.6)
**Inadequate** Number (%)	4 (8.3)	5 (7.2)	2 (5.4)	5 (11.4)	1 (5.3)	-	-	3 (8.5)	7 (5.3)	13 (7.1)

**Table 3:** Inadequate and relatively inadequate items of the Iranian midwifery curriculum based on ICM qualifications

competence adequacy	General competencies	Pre-pregnancy and antenatal care	Care during labor and birth	Ongoing care of women and newborns
**Relatively inadequate**	Acquiring skills in cooperation with women to create and implement a care plan	Gaining knowledge of the necessary care and support for women during and after abortion		Gaining knowledge in the field of providing information to mothers regarding breastfeeding multiple Acquiring knowledge and skills to differentiate postpartum depression from transient anxiety caused by newborn care
**Inadequate**	Acquiring knowledge and skills in the field of self-assessment and reflective practice Acquiring knowledge of personal opinions and their effect on performance. Acquiring skills to update performance by participating in continuing professional education Acquiring skills in the field of midwifery promotion, including participation in national and local associations. Acquiring knowledge and skills regarding the role of midwives as a preceptor, mentor, and role model Acquiring skills in informing women about their rights and responsibilities and the field of midwifery practice Birth facilities and centers and required resources in different places Getting to know the community’s perspective on healthcare facilities and birth centers Critical thinking skills and clinical reasoning in the field of health promotion	Identifying barriers to women’s access and use of reproductive and sexual services and helping to remove the barriers Mobilizing blood donors in case of need. Acquiring knowledge in the field of physical and psychological care and support needed during and after abortion Acquiring counseling skills with women to continue or terminate pregnancy and respect their final decision Providing supportive care in case of continuation of pregnancy Referral to social agencies and services Providing information about abortion procedures, possible complications, and pain management, as well as referrals to abortion service providers upon request, as well as providing post-abortion care.	Acquiring knowledge about cultural and social beliefs and traditions about childbirth	Acquiring skills to support mothers separated from their neonates due to more specialized care Acquiring skills in stabilizing the newborn condition and referring to emergency care centers Acquiring the skill of providing counseling and prevention for mothers and family members who have experienced intrauterine death, neonate death, problems, and congenital anomalies of newborns.

### Delphi results

To cover the inadequate and relatively inadequate cases, the first round of expert recommendations included adding some lessons to the existing curriculum, holding various workshops, and designing new courses (Table 4).

**Table 4:** The final results of the Delphi study

	New lessons	Intended course	Learning area	Agreement percentage in the first round	Agreement percentage in the second round	Agreement percentage in the third round	Recommendations
**1**	Principles of self-assessment and reflective practice	Communication, Health Education and Counseling in Maternal and Child Health and Reproduction (Health 3)	knowledge	55%	66%	88%	Holding a life and communication skills workshop in internship courses should also be considered
**2**	Personal opinions and beliefs and their impact on performance	Communication, Health Education and Counseling in Maternal and Child Health and Reproduction (Health 3)	knowledge	100%			
**3**	Self-management skills related to time management, uncertainty, change, and dealing with stress	Internship in the Field of Management and its Application in Midwifery	skill or behaviors	100%			
**4**	Updating performance through participation in continuing professional education (for example, participation in learning opportunities to apply clinical evidence to improve care such as mortality reviews or policy reviews)	Evidence-Based Midwifery (practical part) - all internships	skill or behaviors	100%			
**5.**	Visiting local and national professional associations and midwifery community	Internship in the Field of Reproductive Health, Mother and Child, and Family Planning – Internship in the Field of Management and its Application in Midwifery	skill or behaviors	55%	66%	88%	Due to the impossibility of this process in all universities, inviting officials and representatives from midwifery associations to visit colleges in universities without these associations to inform students about their work
**6**	The role of midwives as preceptors, mentors, and role models	Communication, Health Education and Counseling in Maternal and Child Health and Reproduction (Health 3)	Knowledge	66%	77%	100%	Patient education should also be considered in this course
**7**	Supporting the growth of the profession through participation in midwifery education in the roles of clinical preceptor, mentor, and role model.	All internships in the field	Skill or behaviors	100%			
**8**	getting women to know the field of midwifery and women’s rights and responsibilities	All internships in the field	Skill or behaviors	100%			Currently, it is done in internships, but it is not included in the curriculum
**9**	Fulfilling the requirements to maintain midwifery registration	All internships in the field	Skill or behaviors	100%			Holding a related skill workshop
**10**	The principles of empowerment	Communication, Health Education and Counseling in Maternal and Child Health and Reproduction (Health 3)	Knowledge	66%	77%	100%	-To be taught as a two-hour session. - Considered as a separate course - To be held in the form of a workshop
**11**	Birth facilities and centers and required resources in different places	Internship in the Field of Management and its Application in Midwifery - Preparation for Childbirth and Physiological Childbirth	Knowledge	100%			Converting the unit “Preparation for childbirth and physiological childbirth” from non-core to core
**12**	Getting to know the community’s perspective on healthcare facilities and birth centers	Preparation for Childbirth and Physiological Childbirth - Internship in the Field of Reproductive Health, Mother and Child and Family Planning	Skill or behaviors	77%	77%	88%	Birth facilities and centers in the country are mostly inactive It is not necessary to consider the traditional birth facilities, but healthcare facilities and birthplaces, even home births can also be considered.
**13**	Critical thinking and clinical reasoning using evidence to promote health	Evidence-based midwifery (practical part) - all internships	Skill or behaviors	100%			Holding a life and communication skills workshop Converting the “Evidence-Based Midwifery” course unit from a non-core to a core unit
**14**	Identifying barriers to women’s access and use of reproductive and sexual services and helping to remove the barriers	Internship in mother and child reproductive health and family planning - Internship in the reproductive health field, mother and child, and family planning	Skill or behaviors	100%			Also should be considered in Gynecological Diseases and Infertility internships
**15**	Developing a care program for women with their participation	All internships in the field	Skill or behaviors	66%	77%	100%	To be taught separately in internships. In pregnancy and childbirth, internships help pregnant women decide about their birth plans, Mother and child internships help women develop a childbearing plan. In gynecologic disease internships, help women develop a middle-aged care plan and screening for breast, cervical, and uterine cancer, etc. The theoretical care plan should be included in the course on Pregnancy and Childbirth 1
**16**	Participatory decision-making in treatments and programs	All internships	Skill or behaviors	100%			The theoretical part of participatory decision-making should be included in Communication, Health Education, and Counseling in Maternal and Child Health and Reproduction (Health 3).
**17**	Mobilization of blood donors in necessary cases	Pregnancy and Childbirth Internships	Skill or behaviors	100%			Adding theoretical content in the course on Midwifery and Reproductive Health in Crises and Disasters
**18**	Getting to know the options and resources available in certain situations; Climatic and geographical limitations, transportation facilities, and available resources in centers	Internship in the Field of Management and its Application in Midwifery	Knowledge	66%	66%	66%	The capacity of our subject is not enough to carry out this program This content was considered unnecessary
**19**	Socio-cultural beliefs and traditions regarding childbirth	Pregnancy and Childbirth (1)	Knowledge	66%	77%	77%	Currently, it is taught in the “Rules and Regulations of Midwifery” course. Competence is considered sufficient
**20**	Breastfeeding counseling in multiple	Internship in maternal and child reproductive health and family planning - Internship in reproductive health, mother and child, and family planning in the field	Skill or behaviors	100%			The multiple breastfeeding theory should be included in the course on Promoting Breastfeeding - It should also be considered in childbirth and postpartum internships
**21**	Differentiating postpartum depression from transient anxiety, assessing access to home help, and providing emotional support to women with postpartum depression	Internship in the Field of Reproductive Health, Mother and Child, and Family Planning	skill or behaviors	100%			It should also be considered in childbirth and postpartum internships
**22**	Support for mothers separated from their neonate who needs special care	Neonatal Internship - Internship in the Field of the Required Neonatal Intensive Care	skill or behaviors	100%			
**23**	Carrying out basic measures for high-risk neonates and transferring them to special care centers	Neonatal Internship - Internships in Normal and Abnormal Delivery (1) and (2)	skill or behaviors	100%			
**24**	Care related to unplanned pregnancies, fetal loss (abortion, intrauterine death, and stillbirth), and pregnancies with fetal abnormalities.	The new course entitled “Pregnancy and Childbirth 5”	Knowledge skill or behaviors	100%			Palliative care for mothers should also be included in this new course – It should be included in relevant guidelines

To address the educational objectives on the care of unplanned pregnancies, fetal loss (abortion, intrauterine death, and stillbirth), and pregnancies with fetal abnormalities, it was proposed that the midwifery curriculum include a new course titled “Pregnancy and Childbirth 5.”

67% (16 lessons) of the proposed lessons in the first round received 100% agreement from the experts in the second round. In the remaining cases, the agreement ranged from 50 to 85%. There was no lesson with less than 50% agreement. All expert members also agreed upon the proposed new course after adding the lesson “Providing palliative methods for women with lost pregnancies or fetal and neonatal death,” which will be presented as a theoretical and practical course.

In the third step, several proposed lessons were provided to the experts for surveying and providing solutions due to the 50 to 85% agreement. The lessons with their justifications were as follows:

- “Visiting local and national professional associations and midwifery community”: insufficient implementation capability in some universities.
- “Principles of women empowerment”: a large amount of content and inadequate capacity of the proposed curriculum to accommodate this content.
- “Remain current in practice by participating in continuing professional education,“ “Principles of self-assessment and reflective practice,“ “Developing a plan of care,” and “Cultural practices and beliefs related to childbearing and reproductive health”: lack of relevance to the course desired to add.
- “Community views about and utilization of health care facilities and place(s) of birth,“ and “Management of options in a specific location”: a lack of requirement.

These lessons achieved more than 85% agreement by including experts’ recommendations, which include:

- Inviting officials and representatives from midwifery associations to visit colleges in universities without these associations to inform students about their work.
- Holding workshops on topics such as empowerment principles and communication techniques to cover some inadequate subjects.
- Learning “Principles of self-assessment and reflective practice” and “Principles of empowerment” in all internships.

Due to the lack of consensus among more than 85% of experts, “Managing the selection of options under special circumstances” was deemed unnecessary. Moreover, according to experts, the lesson entitled “Socio-cultural beliefs and traditions about childbirth,” currently taught in Midwifery Laws and Regulations, was recognized as having adequate competence.

Finally, additional recommendations include converting “Preparation for childbirth and physiological childbirth” and “Evidence-based midwifery” courses from non-core to core status. In addition, it was suggested that the theoretical section of “Breastfeeding counseling for multiples” be added to the course on promoting breastfeeding and “developing a care plan” to the course on pregnancy and childbirth 1.

## Discussion

This study aimed to compare Iran’s midwifery curriculum for a Bachelor’s degree to the revised ICM competencies in 2019. The comparative phase of this study revealed that the midwifery curriculum in Iran is similar to ICM competencies in knowledge at 79.5% and skills and behavior at 71.6%. The category of “Care before and during pregnancy” and the subcategory of “Provide care to women with unintended or mistimed pregnancy” had the most significant number of inadequate cases among the four competency categories classified by ICM. Moreover, “Care during labor and childbirth” was the most compatible category with ICM competencies.

In Iran’s midwifery curriculum, the optimal proportion of practical education to theoretical education has been observed by assigning a practical section to some courses and requiring internships for each theoretical course. When developing new curricula, improving the quality of practical education is also one of the most important factors to consider [3]. The majority of education related to delivering medical services in midwifery and reproductive health is adequately covered in Iran’s midwifery curriculum, which is one of its advantages. In this regard, the courses on “Pregnancy and childbirth”, “Gynecological diseases and infertility”, “Neonatology”, “Children’s diseases”, and “Internal and surgical diseases”, as well as the other related courses in the curriculum, have been effective in equipping students with the knowledge and skills necessary to provide medical care. In courses such as “Complementary and alternative treatments in midwifery” and “Preparation for physiological childbirth,” the necessity of avoiding unnecessary medical interventions during low-risk pregnancy and childbirth for healthy women is emphasized. In addition, the required training is provided in this field.

Teaching research principles and evidence-based practice in both the knowledge and skills areas is an additional strength of the Iranian midwifery curriculum. The core competencies established by the ICM encourage midwives to incorporate the most recent research and recommendations into their clinical practice. To encourage reference to local, national, and international policies, recommendations, and guidelines, the final set of these core competencies and additional competencies (advanced or optional and country-specific) are written non-prescriptive. In addition, some competencies emphasize the responsibility of midwives to stay updated, apply evidence in practice, and be aware of local, national, and international recommendations [21].

The high compliance of Iran’s midwifery curriculum with the international code of ethics for midwives considered by ICM is another strength of Iran’s midwifery curriculum. ICM has established ethical codes in four areas. These areas include midwifery relationships, performance, professional responsibilities, and promotion of midwifery knowledge and performance, each comprising multiple areas [22]. Over the past two decades, medical and paramedical education programs have assumed the responsibility of providing students with fundamental knowledge, including ethical theories and the training and skills necessary to navigate ethical dilemmas [23]. Understanding biomedical ethics is a fundamental component of midwifery care and a required core competency. Moreover, promoting family-centered care and encouraging informed choice and shared decision-making are distinguishing characteristics of midwifery [24].

One of the competencies that Iran’s midwifery curriculum was inadequately specialized to teach was shared decision-making. In addition to the importance of shared decision-making with women and families mentioned in the ICM, the Midwifery Education Accreditation Council (MEAC) in North America considers this competency one of the fundamental competencies [25]. MEAC is a North American organization accrediting midwifery education institutions and programs. A student who graduates from MEAC-accredited programs is eligible to take the national certification examination to become a Certified Professional Midwife (CPM). The fundamental competencies are: “Performance in accordance with professional ethics” and the use of “Shared decision-making with the participation of women and their families,” which enables and supports individuals to make informed health decisions [26]. In Western nations, the objective of midwifery education is to prepare midwives for a joint role in providing care in the hospital and community setting with a high degree of autonomy and continuity of care [27]. These programs promote pregnancy and childbirth as natural processes with a non-interventionist approach for healthy women and effective collaboration with other physicians in cases of complications or concerns [28].

In the midwifery curriculum in Iran, shared decision-making among women, such as developing a care plan, is utilized in all internships. However, based on the consensus of the experts, it was determined that its training should be codified in the midwifery curriculum of Iran in the form of women and families’ participation in care decision-making. Midwife-led continuity of care is the optimal model of delivery care for women of all risk levels, and there is a global imperative to increase access to this model [29]. Midwifery education programs in the United Kingdom, Canada, the United States, New Zealand, Australia, and most of Europe emphasize a woman-centered approach to midwifery care. This approach identifies and responds to each woman’s unique physical, emotional, social, and spiritual needs from the early stages of pregnancy to six weeks after birth [28].

The education of continuous care for women is also a component of the Iranian midwifery curriculum for vaginal childbirth. However, students struggle to fully implement what they have learned and acquire the necessary skills. Studies have identified several factors that can aid in implementing, facilitating, and evaluating continuous care in midwifery education. These factors include a women-centered care model for maternity, empowering midwifery students and women to create a continuous relationship, optimizing the sequence of these experiences in the program, and evaluating students’ performance by service recipients [30].

The current curriculum does not account for clinical preceptor, mentor, and role model participation in midwifery education. Practical and clinical education programs include at least 50% of midwifery education according to the ICM midwifery curriculum [22]. Therefore, the importance of mentors and preceptors, who play a significant role in developing students’ knowledge and practical skills, becomes more evident [31]. Expert members agreed that participation in midwifery education as a clinical preceptor, mentor, and role model should be taught theoretically in the theoretical courses of " Communication, Health Education and Counseling in Maternal and Child Health and Reproduction " and practically in all internships.

Since abortion or any termination of pregnancy in Iran is subject to legal and criminal issues, the care of women with unwanted or unplanned pregnancies is underdeveloped in Iran’s current midwifery curriculum. In Iran, therapeutic abortion requires a referral to the General Department of Legal Medicine for permission. This request must be supported by documents determined by the Iranian judicial system [32]. According to the consensus of the experts, it was decided to include the new course entitled “Pregnancy and Childbirth 5” in the revised curriculum. This course is designed to provide sufficient knowledge and skills regarding care for unplanned pregnancies, fetal loss (abortion, intrauterine fetal death, and stillbirth), and pregnancies with fetal abnormalities. In addition to the courses listed in Table 1, other core and non-core courses are considered and taught in Iran’s midwifery curriculum to meet ICM requirements. These courses include 1- Basic science courses: “Cytology and histology”, “Biochemistry”, “Immunology”, “theoretical microbiology”, “Parasitology, and mycology”; 2- Core courses: “General pathology” and “Specific pathology” and “General pharmacology”; and 3- Non-core courses: “Medical information systems” (information technology in midwifery), theory and practice of “Complementary and alternative treatments in midwifery”, “Traditional medicine and herbal therapy in midwifery”, and “Health economics management”.

According to the consensus of the experts, it was also decided that the courses “Preparation for childbirth and physiological childbirth” and “Evidence-based midwifery” should be changed from non-core to core courses in the revised curriculum. This curriculum will be made public and implemented following the addition of the lessons, and new courses approved by the experts to align it as closely as possible with the ICM-established competencies. The decrease in fertility rate since 2009 in Iran has drawn the attention of politicians to reproduction policies [33]. Considering population policies in Iran, family planning and abortion services and methods lessons which are among the competencies mentioned by ICM will be revised in the Iranian midwifery curriculum. Currently, the issue of promoting childbearing in line with population policies is on the agenda to be applied in the curriculum of midwifery and other disciplines.

Although, there are significant differences between nations in the nature and content of midwifery education programs, including the regulation of performance and influence of professional midwifery associations [34, 35], but specific rules and standards apply commonly to specialized theoretical and clinical education across the countries [36]. The ICM mainly aims at the global promotion of midwifery via training midwives according to the international standards thus, this confederation has suggested that all nations with midwifery education standards adjust their programs to meet these minimal global standards [5]. National assessments of midwifery education and university accreditation systems help to raise standards, particularly in Low and Middle-Income Countries (LMICs) [3]. This study can provide a platform for other countries to set their educational programs with international standards in line with national priorities.

### Strengths and limitations

One of the strengths of the present study is the comparative analysis of the midwifery curriculum in light of all ICM-required standards and competencies across all categories. Furthermore, getting expert opinions, who, in most cases, are members of the midwifery board and from various universities, is another strength of the study. The curriculum for midwives is being revised, and it has not yet reached the implementation phase. Therefore, the non-revised curriculum was utilized in the comparative analysis, which can be considered one of its limitations.

### Application of findings and suggestions

This study provides a reliable assessment to apply appropriate interventions at the ministerial and organizational levels. This is intended to provide more action and support to midwifery policymakers and decision-makers to address the lagging areas of midwifery development. Therefore, this could empower midwives to provide better health care and ultimately contribute to achieving SDGs related to health.

Extensive quantitative and qualitative studies should be conducted on the students and faculty of national and private universities to evaluate the quality of midwifery education based on the revised curriculum and the obstacles to its optimal implementation.

## Conclusion

There was inadequacy or relative inadequacy in the Iranian midwifery curriculum for attaining 24 ICM competencies. In addition to revising Iran’s midwifery curriculum following ICM competencies, it is essential to provide more infrastructure and support to midwifery policymakers to develop and implement midwifery training programs that ensure the training of midwives with adequate competencies for practice.

### Electronic supplementary material

Below is the link to the electronic supplementary material.

Supplementary Material 1

## Data Availability

All data generated or analyzed during this study are included in this published article [and its supplementary information files].

## Abbreviations

ICM: International Confederation of Midwifery
MECA: Midwifery Education Accreditation Council
CPM: Certified Professional Midwife
UNFPA: United Nations Population Fund
SDGs: Sustainable Development Goals
LMICs: Low and Middle-Income Countries
