# Supplementary material for: Mapping of Iranian midwifery curriculum according to the International Confederation of midwives competencies
Source: BMC Med Educ. 2023 Oct 24;23:791. doi: 10.1186/s12909-023-04755-7 (PMC10599037; doi:10.1186/s12909-023-04755-7)
Supplement: Supplementary file 1 — Supplementary Material 1 [file 12909_2023_4755_MOESM1_ESM.docx]

| **1- GENERAL COMPETENCIES** | | | | |
| --- | --- | --- | --- | --- |
| 1.a Assume responsibility for own decisions and actions as an autonomous practitioner | | | | |
| Adequacy | Course number | | Course name | ICM competency |
|  | Non-core | Core |  |  |
| Knowledge | | | | |
| Adequate |  | 22-23 | History, Ethics, Laws and Rights in Midwifery-  Law and Forensic Medicine in Midwifery | Principles of accountability and transparency |
| Adequate |  | 22-23 | History, Ethics, Laws and Rights in Midwifery-  Law and Forensic Medicine in Midwifery | Principles and concepts of autonomy |
| Inadequate |  |  | - | Principles of self-assessment and reflective practice |
| Inadequate |  |  | - | Personal beliefs and their influence on practice |
| Adequate | 61-64 |  | Implementation of a Professional Problem-Solving Plan with a Research Article in Midwifery  Evidence-based Midwifery | Knowledge of evidence-based practices |
| Skills & Behaviors | | | | |
| Relatively adequate |  | 24 | Forensic Medicine Internship in Midwifery | Demonstrate behavior that upholds the public trust in the profession |
| Relatively adequate |  | 82 | Internship in the Field of Management and its Application in Midwifery | Participate in self-evaluation, peer review, and other quality improvement activities |
| Relatively adequate |  | 24 | Forensic Medicine Internship in Midwifery | Balance the responsibility of the midwife to provide the best care with the autonomy of the woman to make her own decisions |
| Relatively adequate |  | 24 | Forensic Medicine Internship in Midwifery | Explain the midwife’s role in providing care that is based on relevant law, ethics, and evidence |
| 1.b Assume responsibility for self-care and self-development as a midwife | | | | |
| Adequacy | Course number | | Course name | ICM competency |
|  | Non-core | Core |  |  |
| Knowledge | | | | |
| Adequate |  | 10-12-15-16 | Practical Microbiology-  Principles of Community Health Services (Health 1)-  Principles and Techniques of Nursing and Midwifery and Working Methods in the  Operating Room and Delivery- Internship in the Principles and Techniques of Nursing and Midwifery and Working Methods in the Operating Room and Delivery | Strategies for managing personal safety, particularly within the facility or community setting |
| Skills & Behaviors | | | | |
| Inadequate |  |  | - | Display skills in the management of self in relation to time management, uncertainty, change and coping with stress |
| Adequate |  | 10-16 | Practical Microbiology  Operating Room and Delivery- Internship in the Principles and Techniques of Nursing and Midwifery and Working Methods in the Operating Room and Delivery | Assume responsibility for personal safety in various practice settings |
| Adequate | 74 | 29-31-33-48-75-79 | Pregnancy and Childbirth 2- Safe and Physiological Natural Childbirth and Methods to Reduce Labor Pain  Pregnancy and Childbirth 3. Abnormal Pregnancy and Childbirth  Pregnancy and Childbirth 4. Physiopathology in Pregnancy and Childbirth  Reproductive, Maternal and Child Health Internship and Family Planning  Midwifery and Reproductive Health in Urban Health  Internship in Normal and Abnormal Pregnancy  Internship in the Field of Reproductive Health, Mother and Child and Family Planning | Maintain up-to-date skills and knowledge concerning protocols, guidelines and safe practice |
| Inadequate |  |  | - | Remain current in practice by participating in continuing professional education (for example, participating in learning opportunities that apply evidence to practice to improve care such as mortality reviews or policy reviews.) |
| Adequate |  | 28-30-32-35-37-39-44-48-54-75-76-77-78-79-80-81-82-83 | All of the internships | Identify and address limitations in personal skill, knowledge, or experience |
| Inadequate |  |  | - | Promote the profession of midwifery, including participation in professional organizations at the local and national level |
| 1.c appropriately delegate aspects of care and provide supervision | | | | |
| Adequacy | Course number | | Course name | ICM competency |
|  | Non-core | Core |  |  |
| Knowledge | | | | |
| Adequate |  | 52-82 | Principles of management and its application in midwifery  Internship in the Field of Management and its Application in Midwifery | Policies and regulation related to delegation |
| Adequate |  | 52-82 | Principles of management and its application in midwifery  Internship in the Field of Management and its Application in Midwifery | Supportive strategies to supervise others |
| Inadequate |  |  | - | Role of midwives as preceptors, mentors, and role models |
| Skills & Behaviors | | | | |
| Adequate |  | 82 | Internship in the Field of Management and its Application in Midwifery | Provide supervision to ensure that practice is aligned with evidence-based clinical practice guidelines |
| Inadequate |  |  | - | Support the profession’s growth through participation in midwifery education in the roles of clinical preceptor, mentor, and role model |
| 1.d Use research to inform practice | | | | |
| Adequacy | Course number | | Course name | ICM competency |
|  | Non-core | Core |  |  |
| Knowledge | | | | |
| Adequate | 57-64 | 17 | Vital statistics  Research method and research plan design  Evidence-based Midwifery | Principles of research and evidence-based practice |
| Adequate |  | 17-19-27 | Vital statistics  Principles of Epidemiology and Control of Diseases (Health 2)  Pregnancy and Childbirth 1- Normal Pregnancy | Epidemiologic concepts relevant to maternal and infant health |
| Adequate | 60-64-72 | 34-45-46 | Neonatology  Communication, Health Education and Counseling in Maternal and Child Health and Reproduction (Health 3)  Maternal, Child and Reproductive Health (Health 4)  Preparation for Childbirth and Physiological Childbirth  Evidence-based Midwifery  Promoting Breastfeeding | Global recommendations for practice and their evidence base (e.g. World Health Organization guidelines) |
| Skills & Behaviors | | | | |
| Relatively adequate | 64 |  | Evidence-based Midwifery | Discuss research findings with women and colleagues |
| Relatively adequate | 61 |  | Implementation of a Professional Problem Solving Plan with a Research Article in Midwifery | Support research in midwifery by participating in the conduct of research |
| 1.e Uphold fundamental human rights of individuals when providing midwifery care | | | | |
| Adequacy | Course number | | Course name | ICM competency |
|  | Non-core | Core |  |  |
| Knowledge | | | | |
| Adequate |  | 22-23-24 | History, Ethics, Laws and Rights in Midwifery-  Law and Forensic Medicine in Midwifery  Forensic Medicine in Midwifery Internship | Laws and/or codes that protect human rights |
| Adequate |  | 22-23-24 | History, Ethics, Laws and Rights in Midwifery-  Law and Forensic Medicine in Midwifery  Forensic Medicine in Midwifery Internship | Sexual, reproductive health rights of women and girls |
| Adequate |  | 50 | Sexual Dysfunction | Development of gender identity and sexual orientation |
| Adequate |  | 22-23-24 | History, Ethics, Laws and Rights in Midwifery-  Law and Forensic Medicine in Midwifery  Forensic Medicine in Midwifery Internship | Principles of ethics and Human Rights within midwifery practice |
| Skills & Behaviors | | | | |
| Relatively adequate |  | 24 | Forensic Medicine in Midwifery Internship | Provide information to women about their sexual and reproductive health rights |
| Inadequate |  |  | - | Inform women about the scope of midwifery practice and women’s rights and responsibilities |
| Relatively adequate |  | 24 | Forensic Medicine in Midwifery Internship | Provide information and support to individuals in complex situations where there are competing ethical principles and rights |
| Adequate |  | 28-30-32-35-37-39-44-48-54-75-76-77-78-79-80-81-82-83 | All of the internships | Practice in accordance with philosophy and code of ethics of the ICM and national standards for health professionals |
| Adequate |  | 28-30-32-35-37-39-44-48-54-75-76-77-78-79-80-81-82-83 | All of the internships | Provide gender sensitive care |
| 1.f Adhere to jurisdictional laws, regulatory requirements, and codes of conduct for midwifery practice | | | | |
| Adequacy | Course number | | Course name | ICM competency |
|  | Non-core | Core |  |  |
| Knowledge | | | | |
| Adequate |  | 22-23-24 | History, Ethics, Laws and Rights in Midwifery-  Law and Forensic Medicine in Midwifery  Forensic Medicine in Midwifery Internship | The laws and regulations of the jurisdiction regarding midwifery |
| Adequate |  | 22-23-24 | History, Ethics, Laws and Rights in Midwifery-  Law and Forensic Medicine in Midwifery  Forensic Medicine in Midwifery Internship | National/state/local community standards of midwifery practice |
| Adequate |  | 22-23-24 | History, Ethics, Laws and Rights in Midwifery-  Law and Forensic Medicine in Midwifery  Forensic Medicine in Midwifery Internship | Ethical principles |
| Relatively adequate | 62 | 22 | History, Ethics, Laws and Rights in Midwifery  Theories and Models of Midwifery and Health | ICM and other midwifery philosophies, values, codes of ethics |
| Skills & Behaviors | | | | |
| Adequate |  | 28-30-32-35-37-39-44-48-54-75-76-77-78-79-80-81-82-83 | All of the internships | Practice according to legal requirements and ethical principles |
| Inadequate |  |  |  | Meet requirements for maintenance of midwifery registration |
| Adequate |  | 28-30-32-35-37-39-44-48-54-75-76-77-78-79-80-81-82-83 | All of the internships | Protect the confidentiality of oral information and written records about the care of women and infants |
| Adequate |  | 28-30-32-35-37-39-44-48-54-75-76-77-78-79-80-81-82-83 | All of the internships | Maintain records of care in the manner required by the health authority |
| Adequate |  | 28-30-32-35-37-39-44-48-54-75-76-77-78-79-80-81-82-83 | All of the internships | Comply with all local reporting regulations for birth and death registration |
| Relatively adequate |  | 24 | Forensic Medicine in Midwifery Internship | Recognize violations of laws, regulations, and ethical codes and take appropriate action |
| Adequate |  | 28-30-32-35-37-39-44-48-54-75-76-77-78-79-80-81-82-83 | All of the internships | Report and document incidents and adverse outcomes as required while providing care |
| 1.g Facilitate women to make individual choices about care | | | | |
| Adequacy | Course number | | Course name | ICM competency |
|  | Non-core | Core |  |  |
| Knowledge | | | | |
| Adequate |  | 18-46-47 | General Psychology, Psychology of Women and Family  Maternal, Child and Reproductive Health (Health 4)  Management and Quality Improvement in Maternal, Child and Reproductive Health (Health 5) | Cultural norms and practices surrounding sexuality, sexual practices, marriage, the childbearing continuum, and parenting |
| Inadequate |  |  |  | Principles of empowerment |
| Adequate | 70 | 12 | Principles of Community Health Services (Health 1)  Education to Patient and Client | Methods of conveying health information to individuals, groups, communities |
| Skills & Behaviors | | | | |
| Adequate |  | 28-30-32-35-37-39-44-48-54-75-76-77-78-79-80-81-82-83 | All of the internships | Advocate for and support women to be the central decision makers in their care |
| Adequate |  | 28-30-32-35-37-39-44-48-54-75-76-77-78-79-80-81-82-83 | All of the internships | Assist women to identify their needs, knowledge, skills, feelings, and preferences throughout the course of care |
| Adequate |  | 48-54-78-79 | Reproductive, Maternal and Child Health Internship and Family Planning  Internship in Gynecological Diseases  Internship in Gynecology and Infertility  Internship in the Field of Reproductive Health, Mother and Child and Family Planning | Provide information and anticipatory guidance about sexual and reproductive health to assist women’s decision making |
| Relatively adequate | 60 |  | Preparation for Childbirth and Physiological Childbirth | Collaborate with women in developing a comprehensive plan of care that respects her preferences and decisions |
| 1.h Demonstrate effective interpersonal communication with women and families, health care teams, and community groups | | | | |
| Adequacy | Course number | | Course name | ICM competency |
|  | Non-core | Core |  |  |
| Knowledge | | | | |
| Adequate |  | 22 | History, Ethics, Laws and Rights in Midwifery | Role and responsibilities of midwives and other maternal – infant health providers |
| Adequate |  | 45 | Communication, Health Education and Counseling in Maternal and Child Health and Reproduction (Health 3) | Principles of effective communication |
| Adequate |  | 47-52-82 | Management and Quality Improvement in Maternal, Child and Reproductive Health (Health 5)  Principles of management and its application in midwifery  Internship in the Field of Management and its Application in Midwifery | Principles of effectively working in health care teams |
| Relatively adequate | 73 | 46 | Maternal, Child and Reproductive Health (Health 4)  Midwifery and Reproductive Health in Crises and Disasters | Cultural practices and beliefs related to childbearing and reproductive health |
| Adequate |  | 49 | Principles of Psychiatry in Midwifery | Principles of communication in crisis situations, e.g. grief and loss, emergencies |
| Skills & Behaviors | | | | |
| Adequate |  | 28-30-32-35-37-39-44-48-54-75-76-77-78-79-80-81-82-83 | All of the internships | Listen to others in an unbiased and empathetic manner |
| Adequate |  | 28-30-32-35-37-39-44-48-54-75-76-77-78-79-80-81-82-83 | All of the internships | Respect one others’ point of view |
| Adequate |  | 28-30-32-35-37-39-44-48-54-75-76-77-78-79-80-81-82-83 | All of the internships | Promote the expression of diverse opinions and perspectives |
| Adequate |  | 28-30-32-35-37-39-44-48-54-75-76-77-78-79-80-81-82-83 | All of the internships | Use the preferred language of the woman or an interpreter to maximize communication |
| Relatively adequate |  | 28-30-32-35-37-39-44-48-54-75-76-77-78-79-80-81-82-83 | All of the internships | Establish ethical and culturally-appropriate boundaries between professional and non-professional relationships |
| Relatively adequate |  | 28-30-32-35-37-39-44-48-54-75-76-77-78-79-80-81-82-83 | All of the internships | Demonstrate cultural sensitivity to women, families, and communities |
| Relatively adequate |  | 28-30-32-35-37-39-44-48-54-75-76-77-78-79-80-81-82-83 | All of the internships | Demonstrate sensitivity and empathy for bereaved women and family members |
| Adequate |  | 28-30-32-35-37-39-44-48-54-75-76-77-78-79-80-81-82-83 | All of the internships | Facilitate teamwork and inter-professional care with other care providers (including students) and community groups/ agencies |
| Relatively adequate |  | 82 | Internship in the Field of Management and its Application in Midwifery | Establish and maintain collaborative relationships with individuals, agencies, institutions that are part of referral networks |
| Adequate |  | 28-30-32-35-37-39-44-48-54-75-76-77-78-79-80-81-82-83 | All of the internships | Convey information accurately and clearly and respond to the needs of individuals |
| 1.i Facilitate normal birth processes in institutional and community settings, including women’s homes | | | | |
| Adequacy | Course number | | Course name | ICM competency |
|  | Non-core | Core |  |  |
| Knowledge | | | | |
| Adequate |  | 18-27-46 | General Psychology, Psychology of Women and Family  Pregnancy and Childbirth 1- Normal Pregnancy  Pregnancy and Childbirth 4. Physiopathology in Pregnancy and Childbirth | Normal biologic, psychologic, social, and cultural aspects of reproduction and early life |
| Adequate |  |  | Pregnancy and Childbirth 1- Normal Pregnancy | Practices that facilitate and those that interfere with normal processes |
| Adequate |  | 12-23-30-46 | Principles of Community Health Services (Health 1)  Law and Forensic Medicine in Midwifery  Normal Delivery Internship  Maternal, Child and Reproductive Health (Health 4) | Policies and protocols about care of women in institutional and community settings |
| Inadequate |  |  | - | Availability of resources in various settings |
| Inadequate |  |  | - | Community views about and utilization of health care facilities and place(s) of birth |
| Skills & Behaviors | | | | |
| Adequate | 60 | 30-76-77 | Normal Delivery Internship  Internships in Normal and Abnormal Delivery (1)  Internships in Normal and Abnormal Delivery (2)  Preparation for Childbirth and Physiological Childbirth | Promote policies and a work culture that values normal birth processes |
| Adequate |  | 28-30-32-35-37-39-44-48-54-75-76-77-78-79-80-81-82-83 | All of the internships | Utilize human and clinical care resources to provide personalized care for women and their infants |
| Adequate |  | 30-76-77 | Normal Delivery Internship  Internships in Normal and Abnormal Delivery (1)  Internships in Normal and Abnormal Delivery (2) | Provide continuity of care by midwives known to woman |
| 1.j Assess the health status, screen for health risks, and promote general health and well-being of women and infants | | | | |
| Adequacy | Course number | | Course name | ICM competency |
|  | Non-core | Core |  |  |
| Knowledge | | | | |
| Adequate |  | 45-46-48 | Communication, Health Education and Counseling in Maternal and Child Health and Reproduction (Health 3)  Maternal, Child and Reproductive Health (Health 4)  Reproductive, Maternal and Child Health Internship and Family Planning | Health needs of women related to reproduction |
| Adequate |  | 33-46 | Pregnancy and Childbirth 4. Physiopathology in Pregnancy and Childbirth  Maternal, Child and Reproductive Health (Health 4) | Health conditions that pose risks during reproduction |
| Adequate |  | 34-35-36-37-46-48-79-80 | Neonatology  Neonatal Internship  Childhood Diseases  Childhood Diseases Internship  Maternal, Child and Reproductive Health (Health 4)  Reproductive, Maternal and Child Health Internship and Family Planning  Internship in the Field of the Required Neonatal Intensive Cares | Health needs of infants and common risks |
| Skills & Behaviors | | | | |
| Relatively adequate |  | 48-54-78-79 | Reproductive, Maternal and Child Health Internship and Family Planning  Internship in Gynecological Diseases  Internship in Gynecology and Infertility  Internship in the Field of Reproductive Health, Mother and Child and Family Planning | Conduct a comprehensive assessment of sexual and reproductive health needs |
| Adequate |  | 48-54-78-79 | Reproductive, Maternal and Child Health Internship and Family Planning  Internship in Gynecological Diseases  Internship in Gynecology and Infertility  Internship in the Field of Reproductive Health, Mother and Child and Family Planning | Assess risk factors and at-risk behavior |
| Adequate |  | 28-30-32-48-54-75-76-78-79 | Normal Pregnancy Internship  Normal Delivery Internship  Normal and Abnormal Delivery Internship  Reproductive, Maternal and Child Health Internship and Family Planning  Internship in Gynecological Diseases  Internship in Normal and Abnormal Pregnancy  Internships in Normal and Abnormal Delivery (1)  Internship in Gynecology and Infertility  Internship in the Field of Health | Order, perform, and interpret laboratory and/ or imaging screening tests |
| Inadequate |  |  | - | Exhibit critical thinking and clinical reasoning informed by evidence when promoting health and well being |
| Adequate |  | 28-30-32-35-37-39-44-48-54-75-76-77-78-79-80-81-82-83 | All of the internships | Provide health information and advice tailored to individual circumstances of women and their families |
| Relatively inadequate |  | 28-30-32-35-37-39-44-48-54-75-76-77-78-79-80-81-82-83 | All of the internships | Collaborate with women to develop and implement a plan of care |
| 1.k Prevent and treat common health problems related to reproduction and early life | | | | |
| Adequacy | Course number | | Course name | ICM competency |
|  | Non-core | Core |  |  |
| Knowledge | | | | |
| Adequate |  | 32-50-53-54-76-78 | Normal and Abnormal Delivery Internship  Sexual Dysfunction  Gynecological Diseases and Infertility  Internship in Gynecological Diseases  Internship in Normal and Abnormal Pregnancy  Internship in Gynecology and Infertility | Common health problems related to sexuality and reproduction |
| Adequate |  | 34-35-80 | Neonatology  Neonatal Internship  Internship in the Field of the Required Neonatal Intensive Cares | Common health problems and deviations from normal of newborn infants |
| Adequate |  | 19-40-41-42-43-44 | Principles of Epidemiology and Control of Diseases (Health 2)  Physiopathology and Internal Diseases (1)  Physiopathology and Internal Diseases (2)  Physiopathology and Internal Diseases (3)  Physiopathology and Surgical Diseases  Internship in Internal and Surgical Diseases | Treatment of common health problems |
| Relatively adequate |  | 19 | Principles of Epidemiology and Control of Diseases (Health 2) | Strategies to prevent and control the acquisition and transmission of environmental and communicable diseases |
| Skills & Behaviors | | | | |
| Adequate |  | 28-30-32-35-37-39-44-48-54-75-76-77-78-79-80-81-82-83 | All of the internships | Maintain/promote safe and hygienic conditions for women and infants |
| Adequate |  | 28-30-32-35-37-39-44-48-54-75-76-77-78-79-80-81-82-83 | All of the internships | Use universal precautions consistently |
| Adequate |  | 28-30-32-48-75-76-77-79 | Normal Pregnancy Internship  Normal Delivery Internship  Normal and Abnormal Delivery Internship  Reproductive, Maternal and Child Health Internship and Family Planning  Internship in Normal and Abnormal Pregnancy  Internship in Normal and Abnormal Delivery (1)  Internship in Normal and Abnormal Delivery (2)  Internship in the Field of Health | Provide options to women for coping with and treating common health problems |
| Adequate |  | 28-30-32-35-37-39-44-48-54-75-76-77-78-79-80-81-82-83 | All of the internships | Use technology and interventions appropriately to promote health and prevent secondary complications |
| Adequate |  | 28-30-32-35-37-39-44-48-54-75-76-77-78-79-80-81-82-83 | All of the internships | Recognize when consultation or referral is indicated for managing identified health problems, including consultation with other midwives |
| Relatively adequate |  | 28-30-32-35-37-39-44-48-54-75-76-77-78-79-80-81-82-83 | All of the internships | Include woman in decision-making about referral to other providers and services |
| 1.l Recognize abnormalities and complications and institute appropriate treatment and referral | | | | |
| Adequacy | Course number | | Course name | ICM competency |
|  | Non-core | Core |  |  |
| Knowledge | | | | |
| Adequate |  | 40-41-42-43-44 | Physiopathology and Internal Diseases (1)  Physiopathology and Internal Diseases (2)  Physiopathology and Internal Diseases (3)  Physiopathology and Surgical Diseases  Internship in Internal and Surgical Diseases | Complications/pathologic conditions related to health status |
| Adequate |  | 31-32-33-40 | Pregnancy and Childbirth 3. Abnormal Pregnancy and Childbirth  Normal and Abnormal Delivery Internship  Pregnancy and Childbirth 4. Physiopathology in Pregnancy and Childbirth  Physiopathology and Internal Diseases (1) | Emergency interventions/life-saving  therapies |
| Adequate |  | 22-40-41-42-43-44 | History, Ethics, Laws and Rights in Midwifery  Physiopathology and Internal Diseases (1)  Physiopathology and Internal Diseases (2)  Physiopathology and Internal Diseases (3)  Physiopathology and Surgical Diseases  Internship in Internal and Surgical Diseases | Limits of midwifery scope of practice and own experience |
| Adequate |  | 12 | Principles of Community Health Services (Health 1) | Available referral systems to access medical and other personnel to manage complications |
| Adequate |  | 12 | Principles of Community Health Services (Health 1) | Community/facility plans and protocols for accessing resources in a timely manner |
| Skills & Behaviors | | | | |
| Adequate |  | 28-30-32-35-37-39-44-48-54-75-76-77-78-79-80-81-82-83 | All of the internships | Maintain up-to-date knowledge, skills, and equipment for responding to emergency situations |
| Adequate |  | 28-30-32-35-37-39-44-48-54-75-76-77-78-79-80-81-82-83 | All of the internships | Recognize situations requiring expertise beyond midwifery care |
| Adequate |  | 28-30-32-35-37-39-44-48-54-75-76-77-78-79-80-81-82-83 | All of the internships | Maintain communication with women about nature of problem, actions taken, and referral if indicated |
| Adequate |  | 28-30-32-35-37-39-44-48-54-75-76-77-78-79-80-81-82-83 | All of the internships | Determine the need for immediate intervention and respond appropriately |
| Adequate |  | 28-30-32-35-37-39-44-48-54-75-76-77-78-79-80-81-82-83 | All of the internships | Implement timely and appropriate intervention, inter-professional consultation and/or timely referral taking account of local circumstances |
| Adequate |  | 28-30-32-35-37-39-44-48-54-75-76-77-78-79-80-81-82-83 | All of the internships | Provide accurate oral and written information to other care providers when referral is made. |
| Adequate |  | 28-30-32-35-37-39-44-48-54-75-76-77-78-79-80-81-82-83 | All of the internships | Collaborate with decision-making if possible and appropriate |
| 1.m Care for women who experience physical and sexual violence and abuse | | | | |
| Adequacy | Course number | | Course name | ICM competency |
|  | Non-core | Core |  |  |
| Knowledge | | | | |
| Adequate | 63 | 18-23-24-46 | General Psychology, Psychology of Women and Family  Law and Forensic Medicine in Midwifery  Forensic Medicine in Midwifery Internship  Maternal, Child and Reproductive Health (Health 4)  Sociology and Social Pathology of Women | Socio-cultural, behavioral, and economic conditions that often accompany violence and abuse |
| Relatively adequate | 63-73 |  | Sociology and Social Pathology of Women  Midwifery and Reproductive Health in Crises and Disasters | Resources in community to assist women and children |
| Adequate |  | 22-23 | History, Ethics, Laws and Rights in Midwifery  Law and Forensic Medicine in Midwifery | Risks of disclosure |
| Skills & Behaviors | | | | |
| Adequate |  | 28-30-32-35-37-39-44-48-54-75-76-77-78-79-80-81-82-83 | All of the internships | Protect privacy and confidentiality |
| Adequate |  | 28-30-32-48-54-75-76-77-78-79 | Normal Pregnancy Internship  Normal Delivery Internship  Normal and Abnormal Delivery Internship  Reproductive, Maternal and Child Health Internship and Family Planning  Internship in Gynecological Diseases  Internship in Normal and Abnormal Pregnancy  Internship in Normal and Abnormal Delivery (1)  Internship in Normal and Abnormal Delivery (2)  Internship in the Field of Health  Internship in Gynecology and Infertility | Provide information to all women about sources of help regardless of whether there is disclosure about violence |
| Adequate |  | 48-79 | Reproductive, Maternal and Child Health Internship and Family Planning  Internship in the Field of Reproductive Health, Mother and Child and Family Planning | Inquire routinely about safety at home, at work |
| Adequate |  | 24-48-79 | Forensic Medicine in Midwifery Internship  Reproductive, Maternal and Child Health Internship and Family Planning  Internship in the Field of Reproductive Health, Mother and Child and Family Planning | Recognize potential signs of abuse from physical appearance, emotional affect, related risk behaviors such as substance abuse |
| Relatively adequate |  | 24 | Forensic Medicine in Midwifery Internship | Provide special support for adolescents and victims of gender-based violence including rape |
| Adequate |  | 28-30-32-35-37-39-44-48-54-75-76-77-78-79-80-81-82-83 | All of the internships | Refer to community resources, assist in locating safe settings as needed |

| **2- PRE-PREGNANCY AND ANTENATAL** | | | | |
| --- | --- | --- | --- | --- |
| 2.a Provide pre-pregnancy care | | | | |
| Adequacy | Course number | | Course name | ICM competency |
|  | Non-core | Core |  |  |
| Knowledge | | | | |
| Adequate | 56-68 | 4-6-27 | anatomy2  physyology2  Pregnancy and Childbirth 1- Normal Pregnancy  Anesthesia and Resuscitation in Midwifery  Reproductive Biology | Anatomy and physiology of female and male related to reproduction and sexual development |
| Relatively adequate |  | 27-46-50 | Pregnancy and Childbirth 1- Normal Pregnancy  Maternal, Child and Reproductive Health (Health 4)  Sexual Dysfunction | Socio-cultural aspects of human sexuality |
| Adequate |  | 27-41-46-53 | Pregnancy and Childbirth 1- Normal Pregnancy  Physiopathology and Internal Diseases (2)  Maternal, Child and Reproductive Health (Health 4)  Gynecological Diseases and Infertility | Evidence-based screening for cancer of reproductive organs and other health problems such as diabetes, hypertension, thyroid conditions, and chronic infections that impact pregnancy |
| Skills & Behaviors | | | | |
| Inadequate |  |  | - | Identify and assist in reducing barriers related to accessing and using sexual and reproductive health services |
| Adequate |  | 48-79 | Reproductive, Maternal and Child Health Internship and Family Planning  Internship in the Field of Reproductive Health, Mother and Child and Family Planning | Assess nutritional status, current immunization status, health behaviors such as smoking, existing medical conditions, and exposure to known teratogens |
| Adequate |  | 54-78 | Internship in Gynecological Diseases  Internship in Gynecology and Infertility | Carry out screening procedures for sexually transmitted and other infections, HIV, cervical cancer |
| Adequate |  | 28-48-75-79 | Normal Pregnancy Internship  Reproductive, Maternal and Child Health Internship and Family Planning  Internship in Normal and Abnormal Pregnancy  Internship in the Field of Reproductive Health, Mother and Child and Family Planning | Provide counseling about nutritional supplements such as folic acid, dietary intake, exercise, updating immunizations as needed, modifying risk behaviors, and prevention of sexually transmitted infections |
| 2.b Determine health status of woman | | | | |
| Adequacy | Course number | | Course name | ICM competency |
|  | Non-core | Core |  |  |
| Knowledge | | | | |
| Adequate |  | 6-28 | Physiology 2  Normal Pregnancy Internship | Physiology of menstrual and ovulatory cycle |
| Adequate |  | 28-75 | Normal Pregnancy Internship  Internship in Normal and Abnormal Pregnancy | Components of a comprehensive health history including psycho-social responses to pregnancy and safety at home |
| Adequate |  | 27-28-38-39 | Pregnancy and Childbirth 1- Normal Pregnancy  Normal Pregnancy Internship  Semiology and Physical Examinations  Semiology and Physical Examination Internship | Components of complete physical exam |
| Adequate |  | 27-28 | Pregnancy and Childbirth 1- Normal Pregnancy  Normal Pregnancy Internship | Health conditions including infections and genetic conditions detected by screening blood and biologic samples |
| Skills & Behaviors | | | | |
| Adequate |  | 28-30-48-75-79 | Normal Pregnancy Internship  Normal Delivery Internship  Reproductive, Maternal and Child Health Internship and Family Planning  Internship in Normal and Abnormal Pregnancy  Internship in the Field of Reproductive Health, Mother and Child and Family Planning | Confirm pregnancy and estimate gestational age from history, physical exam, laboratory test and/or ultrasound |
| Adequate |  | 39-44 | Semiology and Physical Examination Internship  Internship in Internal and Surgical Diseases | Obtain comprehensive health history |
| Adequate |  | 39-44 | Semiology and Physical Examination Internship  Internship in Internal and Surgical Diseases | Perform a complete physical examination |
| Adequate |  | 28-30-32-48-54-75-76-77-78 | Normal Pregnancy Internship  Normal Delivery Internship  Normal and Abnormal Delivery Internship  Reproductive, Maternal and Child Health Internship and Family Planning  Internship in Gynecological Diseases  Internship in Normal and Abnormal Pregnancy  Internships in Normal and Abnormal Delivery (1)  Internships in Normal and Abnormal Delivery (2)  Internship in Gynecology and Infertility | Obtain biologic samples for laboratory tests (e.g. venipuncture, finger puncture, urine samples, and vaginal swabs) |
| Adequate |  | 28-48-54-78-79 | Normal Pregnancy Internship  Reproductive, Maternal and Child Health Internship and Family Planning  Internship in Gynecological Diseases  Internship in Gynecology and Infertility  Internship in the Field of Reproductive Health, Mother and Child and Family Planning | Provide information about conditions that may be detected by screening |
| Adequate |  | 48-79 | Reproductive, Maternal, and Child Health Internship and Family Planning  Internship in the Field of Reproductive Health, Mother and Child, and Family Planning | Assess status of immunizations, and update as indicated |
| Inadequate |  |  | - | Discuss findings and potential implications with woman and mutually determine plan of care |
| 2.c Assess fetal well-being | | | | |
| Adequacy | Course number | | Course name | ICM competency |
|  | Non-core | Core |  |  |
| Knowledge | | | | |
| Adequate |  | 6-25-27-28 | Physiology 2  Embryology  Pregnancy and Childbirth 1- Normal Pregnancy  Normal Pregnancy Internship | Placental physiology, embryology, fetal growth and development, and indicators of fetal well-being |
| Relatively adequate |  | 51-83 | Radiology, Sonology, and Electrology in Midwifery and Women  Internship in Radiology, Sonology, and Electrology in Obstetrics and Gynecology | Evidence-based guidelines for use of ultrasound |
| Skills & Behaviors | | | | |
| Adequate |  | 28-48-75-79 | Normal Pregnancy Internship  Reproductive, Maternal, and Child Health Internship and Family Planning  Internship in Normal and Abnormal Pregnancy  Internship in the Field of Reproductive Health, Mother and Child and Family Planning | Assess fetal size, amniotic fluid volume, fetal position, activity, and heart rate from examination of maternal abdomen |
| Relatively adequate |  | 28-48-75-79 | Normal Pregnancy Internship  Reproductive, Maternal, and Child Health Internship and Family Planning  Internship in Normal and Abnormal Pregnancy  Internship in the Field of Reproductive Health, Mother and Child and Family Planning | Determine whether there are indications for additional assessment/examination and refer accordingly |
| Adequate |  | 28-48-75-79 | Normal Pregnancy Internship  Reproductive, Maternal, and Child Health Internship and Family Planning  Internship in Normal and Abnormal Pregnancy  Internship in the Field of Reproductive Health, Mother and Child and Family Planning | Assess fetal movements and ask woman about fetal activity |
| 2.d Monitor the progression of pregnancy | | | | |
| Adequacy | Course number | | Course name | ICM competency |
|  | Non-core | Core |  |  |
| Knowledge | | | | |
| Adequate |  | 27-40-41-42-43-44 | Pregnancy and Childbirth 1- Normal Pregnancy  Physiopathology and Internal Diseases (1)  Physiopathology and Internal Diseases (2)  Physiopathology and Internal Diseases (3)  Physiopathology and Surgical Diseases  Internship in Internal and Surgical Diseases | Usual physiological and physical changes with advancing pregnancy |
| Adequate |  | 21-27 | Principles of Mother and Child Nutrition  Pregnancy and Childbirth 1- Normal Pregnancy | Nutritional requirements of pregnancy |
| Relatively adequate |  | 49 | Principles of Psychiatry in Midwifery | Common psychological responses to pregnancy and symptoms of psychological distress |
| Adequate |  | 27-28-46-48-75-79 | Pregnancy and Childbirth 1- Normal Pregnancy  Normal Pregnancy Internship  Maternal, Child and Reproductive Health (Health 4)  Reproductive, Maternal and Child Health Internship and Family Planning  Internship in Normal and Abnormal Pregnancy  Internship in the Field of Reproductive Health, Mother and Child and Family Planning | Evidence-informed antenatal care policies and guidelines, including frequency of antenatal visits |
| Skills & Behaviors | | | | |
| Adequate |  | 28-48-75-79 | Normal Pregnancy Internship  Reproductive, Maternal and Child Health Internship and Family Planning  Internship in Normal and Abnormal Pregnancy  Internship in the Field of Reproductive Health, Mother and Child and Family Planning | Conduct assessments throughout pregnancy of woman’s physical and psychological well-being, family relationships, and health education needs |
| Adequate |  | 28-48-75-79 | Normal Pregnancy Internship  Reproductive, Maternal and Child Health Internship and Family Planning  Internship in Normal and Abnormal Pregnancy  Internship in the Field of Reproductive Health, Mother and Child and Family Planning | Provide information regarding normal pregnancy to woman, her partner, family members, or other support persons |
| Adequate |  | 28-48-75-79 | Normal Pregnancy Internship  Reproductive, Maternal and Child Health Internship and Family Planning  Internship in Normal and Abnormal Pregnancy  Internship in the Field of Reproductive Health, Mother and Child and Family Planning | Suggest measures to cope with common discomforts of pregnancy |
| Adequate |  | 28-48-75-76 | Normal Pregnancy Internship  Reproductive, Maternal and Child Health Internship and Family Planning  Internship in Normal and Abnormal Pregnancy  Internships in Normal and Abnormal Delivery (1) | Provide information (including written and/ or pictorial) about danger signs, (e.g. vaginal bleeding, signs of preterm labor, pre-labor, rupture of membranes) emergency preparedness, and when and where to seek help |
| Relatively adequate |  | 28-48-49-75 | Normal Pregnancy Internship  Reproductive, Maternal and Child Health Internship and Family Planning  Internship in Normal and Abnormal Pregnancy | Review findings and revise plan of care with woman as pregnancy progresses |
| 2.e Promote and support health behaviors that improve well being | | | | |
| Adequacy | Course number | | Course name | ICM competency |
|  | Non-core | Core |  |  |
| Knowledge | | | | |
| Adequate |  | 46 | Maternal, Child and Reproductive Health (Health 4) | Impact of adverse social, environmental, and economic conditions on maternal -fetal health |
| Adequate | 55 | 46 | Maternal, Child and Reproductive Health (Health 4)  Nutrition in Maternal and Child Diseases | Effects of inadequate nutrition and heavy physical work |
| Adequate |  | 31 | Pregnancy and Childbirth 3. Abnormal Pregnancy and Childbirth | Effects of tobacco use and exposure to second-hand smoke, use of alcohol and addictive drugs |
| Adequate |  | 14 | Pharmacology (2) | Effects of prescribed medications on fetus |
| Adequate |  | 31-46 | Pregnancy and Childbirth 3. Abnormal Pregnancy and Childbirth  Maternal, Child and Reproductive Health (Health 4) | Community resources for income support, food access, and programs to minimize risks of substance abuse |
| Adequate |  | 19-42 | Principles of Epidemiology and Control of Diseases (Health 2)  Physiopathology and Internal Diseases (3) | Strategies to prevent or reduce risks of mother-to-child disease transmission including infant feeding options for HIV infection |
| Adequate |  | 49 | Principles of Psychiatry in Midwifery | Effects of gender-based violence, emotional abuse, and physical neglect |
| Skills & Behaviors | | | | |
| Adequate |  | 48-79 | Reproductive, Maternal and Child Health Internship and Family Planning  Internship in the Field of Reproductive Health, Mother and Child and Family Planning | Provide emotional support to women to encourage change in health behavior |
| Adequate |  | 48-79 | Reproductive, Maternal and Child Health Internship and Family Planning  Internship in the Field of Reproductive Health, Mother and Child and Family Planning | Provide information to woman and family about impact on mother and fetus of risk conditions |
| Adequate |  | 48-79 | Reproductive, Maternal and Child Health Internship and Family Planning  Internship in the Field of Reproductive Health, Mother and Child and Family Planning | Counsel women about and offer referral to appropriate persons or agencies for assistance and treatment |
| Inadequate |  |  | - | Respect women’s decisions about participating in treatments and programs |
| Adequate |  | 48-79 | Reproductive, Maternal and Child Health Internship and Family Planning  Internship in the Field of Reproductive Health, Mother and Child and Family Planning | Make recommendations and identify resources for smoking reduction/cessation in pregnancy  SIB SYSTEM |
| 2.f Provide anticipatory guidance related to pregnancy, birth, breastfeeding, parenthood, and change in the family | | | | |
| Adequacy | Course number | | Course name | ICM competency |
|  | Non-core | Core |  |  |
| Knowledge | | | | |
| Adequate | 70 | 27-29-45-46 | Pregnancy and Childbirth 1- Normal Pregnancy  Pregnancy and Childbirth 2- Safe and Physiological Natural Childbirth and Methods to Reduce Labor Pain  Communication, Health Education and Counseling in Maternal and Child Health and Reproduction (Health 3)  Maternal, Child and Reproductive Health (Health 4)  Education to Patient and Client | Needs of Individuals and families for different information at different times in their respective life cycles |
| Adequate | 70 | 45 | Communication, Health Education and Counseling in Maternal and Child Health and Reproduction (Health 3)  Education to Patient and Client | Methods of providing information to individuals and groups |
| Adequate |  | 60 | Education to Patient and Client | Methods of eliciting maternal feelings and expectations for self, infant, and family |
| Skills & Behaviors | | | | |
| Adequate |  | 28-48-75-79 | Normal Pregnancy Internship  Reproductive, Maternal and Child Health Internship and Family Planning  Internship in Normal and Abnormal Pregnancy  Internship in the Field of Reproductive Health, Mother and Child and Family Planning | Participate in--and refer women and support persons to--childbirth education programs |
| Adequate |  | 60 | Preparation for Childbirth and Physiological Childbirth | Convey information accurately and clearly and respond to needs of individuals |
| Adequate |  | 60 | Preparation for Childbirth and Physiological Childbirth | Prepare the woman, partner, and family to recognize labor onset, when to seek care, and progress of labor |
| Adequate |  | 76-77-79 | Internships in Normal and Abnormal Delivery (1)  Internships in Normal and Abnormal Delivery (2)  Internship in the Field of Reproductive Health, Mother and Child and Family Planning | Provide information about postpartum needs including contraception, care of newborn infants, and the importance of exclusive breastfeeding for infant health |
| Relatively adequate |  | 28-30-48-75-79 | Normal Pregnancy Internship  Normal Delivery Internship 2  Reproductive, Maternal and Child Health Internship and Family Planning  Internship in Normal and Abnormal Pregnancy  Internship in the Field of Reproductive Health, Mother and Child and Family Planning | Identify needs or problems requiring further expertise or referral such as excessive fear, and dysfunctional relationships |
| 2.g Detect, stabilse, manage, and refer women with complicated pregnancies | | | | |
| Adequacy | Course number | | Course name | ICM competency |
|  | Non-core | Core |  |  |
| Knowledge | | | | |
| Adequate |  | 31 | Pregnancy and Childbirth 3. Abnormal Pregnancy and Childbirth | Complications of early pregnancy such as threatened or actual miscarriage, and ectopic pregnancy |
| Adequate |  | 31 | Pregnancy and Childbirth 3. Abnormal Pregnancy and Childbirth | Fetal compromise, growth restriction, malposition, preterm labor |
| Adequate |  | 31-33 | Pregnancy and Childbirth 3. Abnormal Pregnancy and Childbirth  Pregnancy and Childbirth 4. Physiopathology in Pregnancy and Childbirth | Signs and symptoms of maternal pathologic conditions such as pre-eclampsia, gestational diabetes, and other systemic illnesses |
| Adequate |  | 33 | Pregnancy and Childbirth 4. Physiopathology in Pregnancy and Childbirth | Signs of acute emergencies such as hemorrhage, seizures, and sepsis |
| Skills & Behaviors | | | | |
| Relatively adequate |  | 28-75 | Pregnancy and Childbirth 4. Physiopathology in Pregnancy and Childbirth  Internship in Normal and Abnormal Pregnancy | Stabilize in emergencies and refer for treatment as necessary |
| Adequate |  | 28-75 | Pregnancy and Childbirth 4. Physiopathology in Pregnancy and Childbirth  Internship in Normal and Abnormal Pregnancy | Collaborate in care of complications |
| Adequate |  | 15-16-30-32 | Principles and Techniques of Nursing and Midwifery and Working Methods in the Operating Room and Delivery  Internship in the Principles and Techniques of Nursing and Midwifery and Working Methods in the Operating Room and Delivery  Normal Delivery Internship 2  Normal and Abnormal Delivery Internship | Implement critical care activities to support vital body functions (e.g. intravenous (IV) fluids, magnesium sulphate, antihemorrhagics) |
| Inadequate |  |  | - | Mobilize blood donors if necessary |
| Adequate |  | 75-79 | Internship in Normal and Abnormal Pregnancy  Internship in the Field of Reproductive Health, Mother and Child and Family Planning | Transfer to higher level facility if needed |
| 2.h Assist the woman and her family to plan for an appropriate place of birth | | | | |
| Adequacy | Course number | | Course name | ICM competency |
|  | Non-core | Core |  |  |
| Knowledge | | | | |
| Relatively adequate |  | 29- 60 | Pregnancy and Childbirth 2- Safe and Physiological Natural Childbirth and Methods to Reduce Labor Pain  Preparation for Childbirth and Physiological Childbirth | Evidence about birth outcomes in different birthplace settings |
| Inadequate |  |  | - | Availability of options in a specific location; limitations of climate, geography, means of transport, and resources available in facilities |
| Relatively adequate |  | 29 | Pregnancy and Childbirth 2- Safe and Physiological Natural Childbirth and Methods to Reduce Labor Pain | Local policies and guidelines |
| Skills & Behaviors | | | | |
| Relatively adequate |  | 60 | Preparation for Childbirth and Physiological Childbirth | Discuss options, preferences and contingency plans with woman and support persons and respect their decision |
| Relatively adequate |  | 60 | Preparation for Childbirth and Physiological Childbirth | Provide information about preparing birth site if in community, e.g. travel and admission to facility |
| Relatively adequate |  | 60 | Preparation for Childbirth and Physiological Childbirth | Promote the availability of a full range of birth settings |
| 2.i Provide care to women with unintended or mistimed pregnancy | | | | |
| Adequacy | Course number | | Course name | ICM competency |
|  | Non-core | Core |  |  |
| Knowledge | | | | |
| Inadequate |  |  | - | Complexity of decision-making about unintended or mistimed pregnancies |
| Adequate |  | 46-48-79 | Maternal, Child and Reproductive Health (Health 4)  Reproductive, Maternal and Child Health Internship and Family Planning  Internship in the Field of Reproductive Health, Mother and Child and Family Planning | Emergency contraception |
| Adequate |  | 23 | Law and Forensic Medicine in Midwifery | Legal options for induced abortion; eligibility and availability of medical and surgical abortion service |
| Adequate |  | 23-31 | Law and Forensic Medicine in Midwifery  Pregnancy and Childbirth 3. Abnormal Pregnancy and Childbirth | Medications used to induce abortion; properties, effects, and side effects |
| Adequate |  | 23-31 | Law and Forensic Medicine in Midwifery  Pregnancy and Childbirth 3. Abnormal Pregnancy and Childbirth | Risks of unsafe abortion |
| Adequate |  | 46-48-79 | Maternal, Child and Reproductive Health (Health 4)  Reproductive, Maternal and Child Health Internship and Family Planning  Internship in the Field of Reproductive Health, Mother and Child and Family Planning | Family planning methods appropriate for the post-abortion period |
| Relatively Inadequate |  | 31-32-76-77 | Pregnancy and Childbirth 3. Abnormal Pregnancy and Childbirth  Normal and Abnormal Delivery Internship  Internships in Normal and Abnormal Delivery (1)  Internships in Normal and Abnormal Delivery (1) | Care and support (physical and psychological) needed during and after abortion |
| Skills & Behaviors | | | | |
| Adequate |  | 28-48-75-79 | Normal Pregnancy Internship  Reproductive, Maternal and Child Health Internship and Family Planning  Internship in Normal and Abnormal Pregnancy  Internship in the Field of Reproductive Health, Mother and Child and Family Planning | Confirm pregnancy and determine gestational age; refer for ultrasound if unknown gestation and/or symptoms of ectopic pregnancy |
| Inadequate |  |  | - | Counsel woman about options to maintain or end the pregnancy and respect the ultimate decision |
| Inadequate |  |  | - | Provide supportive antenatal care if the pregnancy continued; refer to agencies, and social services for support and assistance when needed |
| Inadequate |  |  | - | Identify from obstetric, medical and social history, contraindications to medication or aspiration methods |
| Relatively adequate |  | 24 | Forensic Medicine in Midwifery Internship | Provide information about legal regulations, eligibility, and access to abortion services |
| Inadequate |  |  | - | Provide information about abortion procedures, potential complications, management of pain, and when to seek help |
| Inadequate |  |  | - | Refer to provider of abortion services upon request |
| Inadequate |  |  | - | Provide post-abortion care  ̶ Confirm expulsion of products of conception from history, ultrasound, or levels of HCG  ̶ Review options for contraception and initiate immediate use of method  ̶ Explore psychological response to abortion |

| **3- CARE DURING LABOUR AND BIRTH** | | | | |
| --- | --- | --- | --- | --- |
| 3.a Promote physiologic labor and birth | | | | |
| Adequacy | Course number | | Course name | ICM competency |
|  | Non-core | Core |  |  |
| Knowledge | | | | |
| Adequate |  | 4-27-29-56 | Anatomy 2  Pregnancy and Childbirth 1- Normal Pregnancy  Pregnancy and Childbirth 2- Safe and Physiological Natural Childbirth and Methods to Reduce Labor Pain  Anesthesia and Resuscitation in Midwifery | Anatomy of maternal pelvis and fetus; mechanisms of labor for different fetal presentations |
| Adequate |  | 6-29-60 | Physiology 2  Pregnancy and Childbirth 2- Safe and Physiological Natural Childbirth and Methods to Reduce Labor Pain  Preparation for Childbirth and Physiological Childbirth | Physiologic onset and progression of labor |
| Adequate |  | 29 | Pregnancy and Childbirth 2- Safe and Physiological Natural Childbirth and Methods to Reduce Labor Pain | Evidence about interventions in normal labor and birth |
| Inadequate |  |  | - | Cultural and social beliefs and traditions about birth |
| Adequate |  | 29-60 | Pregnancy and Childbirth 2- Safe and Physiological Natural Childbirth and Methods to Reduce Labor Pain  Preparation for Childbirth and Physiological Childbirth | Signs and behaviors of labor progress; factors that impede labor progress |
| Adequate |  | 29 | Pregnancy and Childbirth 2- Safe and Physiological Natural Childbirth and Methods to Reduce Labor Pain | Methods of assessing fetus during labor |
| Skills & Behaviors | | | | |
| Adequate |  | 30-32-76-77 | Normal Delivery Internship  Normal and Abnormal Delivery Internship  Internships in Normal and Abnormal Delivery (1)  Internships in Normal and Abnormal Delivery (2) | Provide care for a woman in the birth setting of her choice, following policies and protocols |
| Adequate |  | 30-32-76-77 | Normal Delivery Internship  Normal and Abnormal Delivery Internship  Internships in Normal and Abnormal Delivery (1)  Internships in Normal and Abnormal Delivery (2) | Obtain relevant obstetric and medical history |
| Adequate |  | 30-32-76-77 | Normal Delivery Internship  Normal and Abnormal Delivery Internship  Internships in Normal and Abnormal Delivery (1)  Internships in Normal and Abnormal Delivery (2) | Perform and interpret focused physical examination of the woman and fetus |
| Adequate |  | 30-32-76-77 | Normal Delivery Internship  Normal and Abnormal Delivery Internship  Internships in Normal and Abnormal Delivery (1)  Internships in Normal and Abnormal Delivery (2) | Order and interpret laboratory tests if needed |
| Adequate |  | 30-32-76-77 | Normal Delivery Internship  Normal and Abnormal Delivery Internship  Internships in Normal and Abnormal Delivery (1)  Internships in Normal and Abnormal Delivery (2) | Assess woman’s physical and behavioral responses to labor |
| Adequate |  | 30-32-76-77 | Normal Delivery Internship  Normal and Abnormal Delivery Internship  Internships in Normal and Abnormal Delivery (1)  Internships in Normal and Abnormal Delivery (2) | Provide information, support, and encouragement to woman and support persons throughout labor and birth |
| Adequate |  | 30-32-76-77 | Normal Delivery Internship  Normal and Abnormal Delivery Internship  Internships in Normal and Abnormal Delivery (1)  Internships in Normal and Abnormal Delivery (2) | Provide respectful one-to-one care |
| Adequate |  | 30-32-76-77 | Normal Delivery Internship  Normal and Abnormal Delivery Internship  Internships in Normal and Abnormal Delivery (1)  Internships in Normal and Abnormal Delivery (2) | Encourage freedom of movement and upright positions |
| Adequate |  | 30-32-76-77 | Normal Delivery Internship  Normal and Abnormal Delivery Internship  Internships in Normal and Abnormal Delivery (1)  Internships in Normal and Abnormal Delivery (2) | Provide nourishment and fluids |
| Adequate |  | 30-32-76-77 | Normal Delivery Internship  Normal and Abnormal Delivery Internship  Internships in Normal and Abnormal Delivery (1)  Internships in Normal and Abnormal Delivery (2) | Offer and support woman to use strategies for coping with labor pain, e.g. controlled breathing, water immersion, relaxation, massage, and pharmacologic modalities when needed |
| Adequate |  | 30-32-76-77 | Normal Delivery Internship  Normal and Abnormal Delivery Internship  Internships in Normal and Abnormal Delivery (1)  Internships in Normal and Abnormal Delivery (2) | Assess regularly parameters of maternal-fetal status, and e.g. vital signs, contractions, cervical changes, and fetal descent |
| Adequate |  | 30-32-76-77 | Normal Delivery Internship  Normal and Abnormal Delivery Internship  Internships in Normal and Abnormal Delivery (1)  Internships in Normal and Abnormal Delivery (2) | Use labour progress graphic display to record findings and assist in detecting complications, e.g. labour delay, fetal compromise, maternal exhaustion, hypertension, infection |
| Adequate |  | 30-32-76-77 | Normal Delivery Internship  Normal and Abnormal Delivery Internship  Internships in Normal and Abnormal Delivery (1)  Internships in Normal and Abnormal Delivery (2) | Augment uterine contractility judiciously using non-pharmacological or pharmacological agents to prevent non-progressive labor |
| Adequate |  | 30-32-76-77 | Normal Delivery Internship  Normal and Abnormal Delivery Internship  Internships in Normal and Abnormal Delivery (1)  Internships in Normal and Abnormal Delivery (2) | Prevent unnecessary routine interventions, e.g. amniotomy, electronic fetal monitoring, directed closed glottis pushing, episiotomy |
| 3.b Manage a safe spontaneous vaginal birth; prevent, detect and stabilize complications | | | | |
| Adequacy | Course number | | Course name | ICM competency |
|  | Non-core | Core |  |  |
| Knowledge | | | | |
| Adequate | 60 | 29-76-77 | Pregnancy and Childbirth 2- Safe and Physiological Natural Childbirth and Methods to Reduce Labor Pain  Preparation for Childbirth and Physiological Childbirth  Internships in Normal and Abnormal Delivery (1)  Internships in Normal and Abnormal Delivery (2) | Manage a safe spontaneous vaginal birth |
| Adequate |  | 29-76-77 | Pregnancy and Childbirth 2- Safe and Physiological Natural Childbirth and Methods to Reduce Labor Pain  Internships in Normal and Abnormal Delivery (1)  Internships in Normal and Abnormal Delivery (2) | Evidence about conduct of third stage, including use of uterotonics |
| Relatively adequate |  | 31-32-76-77 | Pregnancy and Childbirth 3. Abnormal Pregnancy and Childbirth  Normal and Abnormal Delivery Internship  Internships in Normal and Abnormal Delivery (1)  Internships in Normal and Abnormal Delivery (2) | Potential complications and their immediate treatment e.g. shoulder dystocia, and excessive bleeding, fetal compromise, eclampsia, retained placenta |
| Relatively adequate | 56 | 31-32-34-35-76-77-80 | Pregnancy and Childbirth 3. Abnormal Pregnancy and Childbirth  Normal and Abnormal Delivery Internship  Neonatal  Neonatal Internship  Anesthesia and Resuscitation in Midwifery  Internships in Normal and Abnormal Delivery (1)  Internships in Normal and Abnormal Delivery (2)  Internship in the Field of the Required Neonatal Intensive Cares | Management of emergencies as covered in emergency skills training programmes  such as BEmONC, HMS |
| Adequate |  | 29-76-77 | Pregnancy and Childbirth 2- Safe and Physiological Natural Childbirth and Methods to Reduce Labor Pain  Internships in Normal and Abnormal Delivery (1)  Internships in Normal and Abnormal Delivery (2) | Signs of placental separation; appearance of normal placenta, membranes, and umbilical cord |
| Adequate |  | 31-32-76-77 | Pregnancy and Childbirth 3. Abnormal Pregnancy and Childbirth  Normal and Abnormal Delivery Internship  Internships in Normal and Abnormal Delivery (1)  Internships in Normal and Abnormal Delivery (2) | Types of perineal and vaginal trauma requiring repair and suturing techniques |
| Skills & Behaviors | | | | |
| Adequate |  | 30-32-76-77 | Normal Delivery Internship  Normal and Abnormal Delivery Internship  Internships in Normal and Abnormal Delivery (1)  Internships in Normal and Abnormal Delivery (2) | Support the woman to give birth in her position of choice |
| Adequate |  | 30-32-76-77 | Normal Delivery Internship  Normal and Abnormal Delivery Internship  Internships in Normal and Abnormal Delivery (1)  Internships in Normal and Abnormal Delivery (2) | Ensure clean environment, presence of clean necessary supplies and source of warmth |
| Adequate |  | 30-32-76-77 | Normal Delivery Internship  Normal and Abnormal Delivery Internship  Internships in Normal and Abnormal Delivery (1)  Internships in Normal and Abnormal Delivery (2) | Coach woman about pushing to control expulsion of presenting part, avoid routine episiotomy |
| Relatively adequate |  | 30-32-76-77 | Normal Delivery Internship  Normal and Abnormal Delivery Internship  Internships in Normal and Abnormal Delivery (1)  Internships in Normal and Abnormal Delivery (2) | Undertake appropriate maneuvers and use maternal position to facilitate vertex, face, or breech birth |
| Adequate |  | 30-32-76-77 | Normal Delivery Internship  Normal and Abnormal Delivery Internship  Internships in Normal and Abnormal Delivery (1)  Internships in Normal and Abnormal Delivery (2) | Expedite birth in presence of fetal distress |
| Adequate |  | 30-32-76-77 | Normal Delivery Internship  Normal and Abnormal Delivery Internship  Internships in Normal and Abnormal Delivery (1)  Internships in Normal and Abnormal Delivery (2) | Delay cord clamping |
| Adequate |  | 30-32-76-77 | Normal Delivery Internship  Normal and Abnormal Delivery Internship  Internships in Normal and Abnormal Delivery (1)  Internships in Normal and Abnormal Delivery (2) | Manage nuchal cord |
| Adequate |  | 30-32-76-77 | Normal Delivery Internship  Normal and Abnormal Delivery Internship  Internships in Normal and Abnormal Delivery (1)  Internships in Normal and Abnormal Delivery (2) | Assess immediate condition of newborn |
| Adequate |  | 30-32-76-77 | Normal Delivery Internship  Normal and Abnormal Delivery Internship  Internships in Normal and Abnormal Delivery (1)  Internships in Normal and Abnormal Delivery (2) | Provide skin to skin contact and warm environment |
| Adequate |  | 30-32-76-77 | Normal Delivery Internship  Normal and Abnormal Delivery Internship  Internships in Normal and Abnormal Delivery (1)  Internships in Normal and Abnormal Delivery (2) | Deliver placenta and membranes and inspect for completeness |
| Adequate |  | 30-32-76-77 | Normal Delivery Internship  Normal and Abnormal Delivery Internship  Internships in Normal and Abnormal Delivery (1)  Internships in Normal and Abnormal Delivery (2) | Assess uterine tone, maintain firm contraction, and estimate and record maternal blood loss; manage excessive blood loss including administration of uterotonics |
| Adequate |  | 30-32-76-77 | Normal Delivery Internship  Normal and Abnormal Delivery Internship  Internships in Normal and Abnormal Delivery (1)  Internships in Normal and Abnormal Delivery (2) | Inspect vaginal and perineal areas for trauma, and repair as needed, following policies and protocols |
| Relatively adequate |  | 30-32-76-77 | Normal Delivery Internship  Normal and Abnormal Delivery Internship  Internships in Normal and Abnormal Delivery (1)  Internships in Normal and Abnormal Delivery (2) | Provide first line measures to treat or stabilize identified conditions |
| Relatively adequate |  | 30-32-76-77 | Normal Delivery Internship  Normal and Abnormal Delivery Internship  Internships in Normal and Abnormal Delivery (1)  Internships in Normal and Abnormal Delivery (2) | Refer for continuing treatment of any complications as needed |
| 3.c Provide care of the newborn immediately after birth | | | | |
| Adequacy | Course number | | Course name | ICM competency |
|  | Non-core | Core |  |  |
| Knowledge | | | | |
| Adequate |  | 29-30 | Pregnancy and Childbirth 2- Safe and Physiological Natural Childbirth and Methods to Reduce Labor Pain  Normal Delivery Internship | Normal transition to extra-uterine environment |
| Adequate |  | 29-34-35 | Pregnancy and Childbirth 2- Safe and Physiological Natural Childbirth and Methods to Reduce Labor Pain  Neonatology  Neonatology Internship | Scoring systems to assess newborn status |
| Adequate |  | 29-30 | Pregnancy and Childbirth 2- Safe and Physiological Natural Childbirth and Methods to Reduce Labor Pain  Normal Delivery Internship | Signs indicating need for immediate actions to assist transition |
| Relatively adequate |  | 29-34-35 | Pregnancy and Childbirth 2- Safe and Physiological Natural Childbirth and Methods to Reduce Labor Pain  Neonatology  Neonatology Internship | Interventions to establish breathing and circulation as covered in training programs such as HBS |
| Adequate |  | 29-30-34-35 | Pregnancy and Childbirth 2- Safe and Physiological Natural Childbirth and  Methods to Reduce Labor Pain  Normal Delivery Internship  Neonatology  Neonatology Internship | Appearance and behavior of healthy newborn infant |
| Adequate |  | 29-30-34-35 | Pregnancy and Childbirth 2- Safe and Physiological Natural Childbirth and Methods to Reduce Labor Pain  Normal Delivery Internship  Neonatology  Neonatology Internship | Method of assessing gestational age of newborn infant |
| Adequate |  | 34-35-80 | Neonatology  Neonatology Internship  Internship in the Field of the Required Neonatal Intensive Cares | Needs of small for gestational age and low birth weight infants |
| Skills & Behaviors | | | | |
| Adequate |  | 30-32-35-76-77 | Normal Delivery Internship  Normal and Abnormal Delivery Internship  Neonatology Internship  Internships in Normal and Abnormal Delivery (1)  Internships in Normal and Abnormal Delivery (2) | Use standardized method to assess newborn condition in the first minutes of life (Apgar or other); refer if needed |
| Adequate |  | 30-32-35- 76-77 | Normal Delivery Internship  Normal and Abnormal Delivery Internship  Neonatology Internship  Internships in Normal and Abnormal Delivery (1)  Internships in Normal and Abnormal Delivery (2) | Institute actions to support breathing and oxygenation |
| Adequate |  | 30-32-35-76-77 | Normal Delivery Internship  Normal and Abnormal Delivery Internship  Neonatology Internship  Internships in Normal and Abnormal Delivery (1)  Internships in Normal and Abnormal Delivery (2) | Provide a safe warm environment for initiating breastfeeding and attachment (bonding) in the first hour of life |
| Adequate |  | 30-32-35-76-77 | Normal Delivery Internship  Normal and Abnormal Delivery Internship  Neonatology Internship  Internships in Normal and Abnormal Delivery (1)  Internships in Normal and Abnormal Delivery (2) | Conduct a complete physical examination of newborn in presence of mother/family; explain findings and expected changes e.g. color of extremities, molding of head. Refer for abnormal findings. |
| Adequate |  | 30-32-35-76-77 | Normal Delivery Internship  Normal and Abnormal Delivery Internship  Neonatology Internship  Internships in Normal and Abnormal Delivery (1)  Internships in Normal and Abnormal Delivery (2) | Institute newborn prophylaxis e.g. ophthalmic infection, and hemorrhagic disease, according to policies and guidelines |
| Adequate |  | 30-32-35-76-77 | Normal Delivery Internship  Normal and Abnormal Delivery Internship  Neonatology Internship  Internships in Normal and Abnormal Delivery (1)  Internships in Normal and Abnormal Delivery (2) | Promote care by mother, frequent feeding and close observation |
| Adequate |  | 30-33-76-77 | Normal Delivery Internship  Normal and Abnormal Delivery Internship  Neonatology Internship  Internships in Normal and Abnormal Delivery (1)  Internships in Normal and Abnormal Delivery (2) | Involve partner/support persons in providing newborn care |

| **4- ONGOING CARE OF WOMEN AND NEWBORNS** | | | | |
| --- | --- | --- | --- | --- |
| 4.a Provide postnatal care for the healthy woman | | | | |
| Adequacy | Course number | | Course name | ICM competency |
|  | Non-core | Core |  |  |
| Knowledge | | | | |
| Adequate |  | 46-48-79 | Maternal, Child and Reproductive Health (Health 4)  Reproductive, Maternal and Child Health Internship and Family Planning  Internship in the Field of Reproductive Health, Mother and Child and Family Planning | Physiological changes following birth, uterine involution, onset of lactation, healing of perineal-vaginal tissues |
| Adequate |  | 29-30-46-48-79 | Pregnancy and Childbirth 2- Safe and Physiological Natural Childbirth and Methods to Reduce Labor Pain  Normal Delivery Internship  Maternal, Child and Reproductive Health (Health 4)  Reproductive, Maternal and Child Health Internship and Family Planning  Internship in the Field of Reproductive Health, Mother and Child and Family Planning | Common discomforts of the postnatal period and comfort measures |
| Adequate | 55-60 | 71 | Nutrition in Maternal and Child Diseases  Preparation for Childbirth and Physiological Childbirth  Nutrition before, during and after Pregnancy | Need for rest, support, and nutrition to support lactation |
| Adequate | 60 | 18-49 | General Psychology, Psychology of Women and Family  Principles of Psychiatry in Midwifery  Preparation for Childbirth and Physiological Childbirth | Psychological responses to mothering role, addition of infant to family |
| Skills & Behaviors | | | | |
| Adequate |  | 32-76-77 | Normal and Abnormal Delivery Internship  Internships in Normal and Abnormal Delivery (1)  Internships in Normal and Abnormal Delivery (2) | Review history of pregnancy, labor, and birth |
| Adequate |  | 32-76-77 | Normal and Abnormal Delivery Internship  Internships in Normal and Abnormal Delivery (1)  Internships in Normal and Abnormal Delivery (2) | Conduct a focused physical exam to assess breast changes and involution. Monitor blood loss and other body functions |
| Adequate |  | 48-79 | Reproductive, Maternal and Child Health Internship and Family Planning  Internship in the Field of Reproductive Health, Mother and Child and Family Planning | Assess mood and feelings about motherhood and demands of infant care |
| Adequate |  | 32-76-77 | Normal and Abnormal Delivery Internship  Internships in Normal and Abnormal Delivery (1)  Internships in Normal and Abnormal Delivery (2) | Provide pain control strategies if needed for uterine contractions, and perineal trauma |
| Adequate |  | 32-76-77 | Normal and Abnormal Delivery Internship  Internships in Normal and Abnormal Delivery (1)  Internships in Normal and Abnormal Delivery (2) | Provide information about self-care that enables mother to meet needs of newborn, e.g. adequate food, nutritional supplements, usual activities, rest periods, and household help |
| Adequate |  | 32-76-77 | Normal and Abnormal Delivery Internship  Internships in Normal and Abnormal Delivery (1)  Internships in Normal and Abnormal Delivery (2) | Provide information about safe sex, family planning methods appropriate for the immediate postnatal period, and pregnancy spacing |
| 4.b Provide care to healthy newborn infant | | | | |
| Adequacy | Course number | | Course name | ICM competency |
|  | Non-core | Core |  |  |
| Knowledge | | | | |
| Adequate |  | 34-35-80 | Neonatology  Neonatology Internship  Internship in the Field of the Required Neonatal Intensive Care | Appearance and behavior of infant in early life; cardio-respiratory changes related to adapting to extra-uterine life |
| Adequate | 74 | 34-35-46-48-79 | Neonatology  Neonatology Internship  Maternal, Child and Reproductive Health (Health 4)  Reproductive, Maternal and Child Health Internship and Family Planning  Midwifery and Reproductive Health in Urban Health  Internship in the Field of Reproductive Health, Mother and Child and Family Planning | Growth and development in initial weeks and months of life |
| Adequate |  | 36-46 | Childhood Diseases  Maternal, Child and Reproductive Health (Health 4) | Protocols for screening for metabolic conditions, infectious conditions, and congenital abnormalities |
| Adequate |  | 34-46-48-79 | Neonatology  Maternal, Child and Reproductive Health (Health 4)  Reproductive, Maternal and Child Health Internship and Family Planning  Internship in the Field of Reproductive Health, Mother and Child and Family Planning | Protocols/guidelines for immunizations in infancy |
| Relatively adequate |  | 46 | Maternal, Child and Reproductive Health (Health 4) | Evidence-based information about infant circumcision; family values, beliefs, and cultural norms |
| Skills & Behaviors | | | | |
| Adequate |  | 48-79 | Reproductive, Maternal and Child Health Internship and Family Planning  Internship in the Field of Reproductive Health, Mother and Child and Family Planning | Examine infant at frequent intervals to monitor growth and developmental behavior |
| Adequate |  | 35-80 | Neonatology Internship  Internship in the Field of the Required Neonatal Intensive Care | Distinguish normal variation in newborn appearance and behavior from those indicating pathologic conditions |
| Adequate |  | 35-48-79-80 | Neonatology Internship  Reproductive, Maternal and Child Health Internship and Family Planning  Internship in the Field of Reproductive Health, Mother and Child and Family Planning  Internship in the Field of the Required Neonatal Intensive Care | Administer immunizations, carry out screening tests as indicated |
| Adequate |  | 35-48-79-80 | Neonatology Internship  Reproductive, Maternal and Child Health Internship and Family Planning  Internship in the Field of Reproductive Health, Mother and Child and Family Planning  Internship in the Field of the Required Neonatal Intensive Care | Provide information to parents about a safe environment for infant, frequent feeding, care of umbilical cord, voiding and stooling, and close physical contact |
| 4.c Promote and support breastfeeding | | | | |
| Adequacy | Course number | | Course name | ICM competency |
|  | Non-core | Core |  |  |
| Knowledge | | | | |
| Adequate | 72 | 6 | Physiology 2  Promoting Breastfeeding | Physiology of lactation |
| Adequate | 72 | 34-35-46 | Neonatology  Neonatology Internship  Maternal, Child and Reproductive Health (Health 4)  Promoting Breastfeeding | Nutritional needs of newborn infants, including low birth weight infants |
| Relatively adequate |  | 46 | Maternal, Child and Reproductive Health (Health 4) | Social, psychological, and cultural aspects of breastfeeding |
| Adequate | 71-72 | 21-46 | Principles of Mother and Child Nutrition  Maternal, Child and Reproductive Health (Health 4)  Nutrition before, during and after Pregnancy  Promoting Breastfeeding | Evidence about benefits of breastfeeding |
| Adequate |  | 14 | Pharmacology (2) | Indications and contraindications to use of drugs and substances during lactation |
| Adequate | 72 | 46 | Maternal, Child and Reproductive Health (Health 4)  Promoting Breastfeeding | Awareness of lactation aids |
| Skills & Behaviors | | | | |
| Adequate |  | 30-48-79 | Normal Delivery Internship  Reproductive, Maternal and Child Health Internship and Family Planning  Internship in the Field of Reproductive Health, Mother and Child and Family Planning | Promote early and exclusive breastfeeding while respecting a woman’s choice regarding newborn feeding |
| Adequate |  | 30-48-79 | Normal Delivery Internship  Reproductive, Maternal and Child Health Internship and Family Planning  Internship in the Field of Reproductive Health, Mother and Child and Family Planning | Provide information about infant needs, frequency and duration of feedings, and weight gain |
| Adequate | 72 | 48-79 | Reproductive, Maternal and Child Health Internship and Family Planning  Promoting Breastfeeding  Internship in the Field of Reproductive Health, Mother and Child and Family Planning | Provide support and information about breastfeeding for a minimum of six months, including combining with work, maintaining milk supply, and storing breast milk |
| Adequate | 72 | 48-79 | Reproductive, Maternal and Child Health Internship and Family Planning  Promoting Breastfeeding  Internship in the Field of Reproductive Health, Mother and Child and Family Planning | Identify and manage breastfeeding problems (e.g. mastitis, low milk supply, engorgement, improper latch) |
| Relatively inadequate | 72 | 48-79 | Reproductive, Maternal and Child Health Internship and Family Planning  Promoting Breastfeeding  Internship in the Field of Reproductive Health, Mother and Child and Family Planning | Provide information to women breastfeeding multiple newborns |
| Adequate | 72 | 48-79 | Reproductive, Maternal and Child Health Internship and Family Planning  Promoting Breastfeeding  Internship in the Field of Reproductive Health, Mother and Child and Family Planning | Refer women to breastfeeding support as indicated |
| Adequate | 72 | 48-79 | Reproductive, Maternal and Child Health Internship and Family Planning  Promoting Breastfeeding  Internship in the Field of Reproductive Health, Mother and Child and Family Planning | Advocate for breastfeeding in family and community |
| 4.d Detect, treat, and stabilize postnatal complications in woman and refer as necessary | | | | |
| Adequacy | Course number | | Course name | ICM competency |
|  | Non-core | Core |  |  |
| Knowledge | | | | |
| Adequate |  | 29-30 | Pregnancy and Childbirth 2- Safe and Physiological Natural Childbirth and Methods to Reduce Labor Pain  Normal Delivery Internship | Signs and symptoms of:  ̶ conditions in the postnatal period that may respond to early intervention (e.g. sub-involution, anemia, and urinary retention)  ̶ complications that need referral to more specialized provider or facility (e.g. hematoma, thrombophlebitis, sepsis, obstetric fistula, and incontinence)  ̶ life threatening complications requiring immediate response and specialized care (hemorrhage, amniotic fluid embolus, seizure, and stroke) |
| Relatively adequate |  | 49 | Principles of Psychiatry in Midwifery | Signs and symptoms of postnatal depression, anxiety, and psychosis |
| Relatively adequate |  | 49 | Principles of Psychiatry in Midwifery | Mourning process following perinatal death |
| Skills & Behaviors | | | | |
| Adequate |  | 76-77 | Abnormal Delivery (1)  Internships in Normal and Abnormal Delivery (2) | Provide information to woman and family about potential complications and when to seek help |
| Adequate |  | 48-76-77-79 | Reproductive, Maternal and Child Health Internship and Family Planning  Internships in Normal and Abnormal Delivery (1)  Internships in Normal and Abnormal Delivery (2)  Internship in the Field of Reproductive Health, Mother and Child and Family Planning | Assess woman during postnatal period to detect signs and symptoms of complications |
| Relatively inadequate |  | 48-76-77-79 | Reproductive, Maternal and Child Health Internship and Family Planning  Internships in Normal and Abnormal Delivery (1)  Internships in Normal and Abnormal Delivery (2)  Internship in the Field of Reproductive Health, Mother and Child and Family Planning | Distinguish postnatal depression from transient anxiety about caring for baby, assess availability of help and support at home, and provide emotional support |
| Inadequate |  |  | - | Provide counseling and follow-up care for women and family members who experience stillbirth, neonatal death, serious infant illness, and congenital conditions |
| Adequate |  | 30-32-48-79 | Normal Delivery Internship  Normal and Abnormal Delivery Internship  Reproductive, Maternal and Child Health Internship and Family Planning  Internship in the Field of Reproductive Health, Mother and Child and Family Planning | Provide first line measures to treat or stabilize identified conditions |
| Adequate |  | 30-32-48-79 | Normal Delivery Internship  Normal and Abnormal Delivery Internship  Reproductive, Maternal and Child Health Internship and Family Planning  Internship in the Field of Reproductive Health, Mother and Child and Family Planning | Arrange referral and/or transfer as needed |
| 4.e Detect, stabilize, and manage health problems in newborn infant and refer if necessary | | | | |
| Adequacy | Course number | | Course name | ICM competency |
|  | Non-core | Core |  |  |
| Knowledge | | | | |
| Adequate |  | 25-26-34-35-80 | Embryology  Genetics  Neonatal    Neonatal Internship  Internship in the Field of Required Neonatal Intensive Care | Congenital anomalies, and genetic conditions |
| Adequate |  | 34-35-80 | Neonatal    Neonatal Internship  Internship in the Field of Required Neonatal Intensive Care | Needs of pre-term and low birth weight infants |
| Adequate |  | 31 | Pregnancy and Childbirth 3. Abnormal Pregnancy and Childbirth | Symptoms and treatment of withdrawal from maternal drug use |
| Adequate |  | 33 | Pregnancy and Childbirth 4. Physiopathology in Pregnancy and Childbirth | Prevention of mother-to-child transmission of infections such as HIV, hepatitis B and C |
| Adequate |  | 34 | Neonatology | Signs and symptoms of common health problems and complications; their immediate and ongoing treatment |
| Skills & Behaviors | | | | |
| Adequate |  | 35-80 | Neonatal Internship  Internship in the Field of Required Neonatal Intensive Care | Assess and recognize abnormal findings |
| Adequate |  | 35-80 | Neonatal Internship  Internship in the Field of Required Neonatal Intensive Care | Implement protocols for care of low birth weight infants, e.g. ensure warmth, nutrition, monitor condition, “Kangaroo” care as appropriate |
| Adequate | 70 | 35-80 | Neonatal Internship  Education to Patient and Client  Internship in the Field of Required Neonatal Intensive Care | Provide information to mother and support persons about infant condition |
| Inadequate |  |  | - | Provide support in situations where infant separated from mother for special care |
| Adequate |  | 35-80 | Neonatal Internship  Internship in the Field of Required Neonatal Intensive Care | Recognize indications of the need for specialized care in the newborn |
| Inadequate |  |  | - | Stabilize and transfer the at-risk newborn to emergency care facility |
| 4.f Provide family planning services | | | | |
| Adequacy | Course number | | Course name | ICM competency |
|  | Non-core | Core |  |  |
| Knowledge | | | | |
| Adequate | 68 | 4-6-27 | Anatomy 2  Physiology 2  Pregnancy and Childbirth 1- Normal Pregnancy  Reproductive Biology | Anatomy and physiology of female and male related to reproduction and sexual development |
| Relatively adequate |  | 50 | Sexual Dysfunction | Socio-cultural aspects of human sexuality |
| Adequate |  | 46-48 | Maternal, Child and Reproductive Health (Health 4)  Reproductive, Maternal and Child Health Internship and Family Planning | Family planning methods including natural, barrier, hormonal, implantable; emergency contraception, sterilization; their possible side effects, risk of pregnancy, and contraindications to use |
| Adequate |  | 29-46-48 | Pregnancy and Childbirth 2- Safe and Physiological Natural Childbirth and Methods to Reduce Labor Pain  Maternal, Child and Reproductive Health (Health 4)  Reproductive, Maternal and Child Health Internship and Family Planning | Available written and pictorial resources for teaching about family planning methods |
| Adequate |  | 42 | Physiopathology and Internal Diseases (3) | Pregnancy options for HIV positive women or couples |
| Skills & Behaviors | | | | |
| Adequate |  | 48-79 | Reproductive, Maternal and Child Health Internship and Family Planning  Internship in the Field of Reproductive Health, Mother and Child and Family Planning | Provide and protect privacy and confidentiality for discussions about family planning knowledge, goals for limiting and/or spacing of children, and concerns and myths about methods |
| Relatively adequate |  | 48-79 | Reproductive, Maternal and Child Health Internship and Family Planning  Internship in the Field of Reproductive Health, Mother and Child and Family Planning | Obtain relevant history of use of methods, medical conditions, sociocultural values, and preferences that influence choice of method |
| Adequate |  | 48-79 | Reproductive, Maternal and Child Health Internship and Family Planning  Internship in the Field of Reproductive Health, Mother and Child and Family Planning | Provide information about how to use, effectiveness, and cost of various methods to support informed decision-making |
| Adequate |  | 48-79 | Reproductive, Maternal and Child Health Internship and Family Planning  Internship in the Field of Reproductive Health, Mother and Child and Family Planning | Provide methods according to scope of practice and protocols, or refer to another provider |
| Adequate |  | 48-79 | Reproductive, Maternal and Child Health Internship and Family Planning  Internship in the Field of Reproductive Health, Mother and Child and Family Planning | Provide follow-up assessment of use, satisfaction, and side-effects |
| Adequate |  | 48-79 | Reproductive, Maternal and Child Health Internship and Family Planning  Internship in the Field of Reproductive Health, Mother and Child and Family Planning | Refer for woman or partner for sterilization procedure |
